# Cross-reactive CD8^+^ T cell responses to tumor-associated antigens (TAAs) and homologous microbiota-derived antigens (MoAs)

**DOI:** 10.1186/s13046-024-03004-z

**Published:** 2024-03-20

**Authors:** Beatrice Cavalluzzo, Marie Christine Viuff, Siri Amanda Tvingsholm, Concetta Ragone, Carmen Manolio, Angela Mauriello, Franco M. Buonaguro, Maria Lina Tornesello, Francesco Izzo, Alessandro Morabito, Sine Reker Hadrup, Maria Tagliamonte, Luigi Buonaguro

**Affiliations:** 1 Innovative Immunological Models Unit, Istituto Nazionale Tumori - IRCCS - “Fond G. Pascale”, Via Mariano Semmola, 52, Naples, Italy; 2 Department of Health Technology, Section of Experimental and Translational Immunology, Technical University of Denmark, Kongens Lyngby, Denmark; 3 Molecular Biology and Viral Oncogenesis Unit, Istituto Nazionale Tumori - IRCCS - “Fond G. Pascale”, Naples, Italy; 4 Hepatobiliary Surgical Oncology Unit, Istituto Nazionale Tumori - IRCCS - “Fond. G. Pascale”, Naples, Italy; 5 Thoracic Medical Oncology, Istituto Nazionale Tumori - IRCCS - “Fond G. Pascale”, Naples, Italy

## Abstract

**Background:**

We have recently shown extensive sequence and conformational homology between tumor-associated antigens (TAAs) and antigens derived from microorganisms (MoAs). The present study aimed to assess the breadth of T-cell recognition specific to MoAs and the corresponding TAAs in healthy subjects (HS) and patients with cancer (CP).

**Method:**

A library of > 100 peptide-MHC (pMHC) combinations was used to generate DNA-barcode labelled multimers. Homologous peptides were selected from the Cancer Antigenic Peptide Database, as well as Bacteroidetes/Firmicutes-derived peptides. They were incubated with CD8 + T cells from the peripheral blood of HLA-A*02:01 healthy individuals (*n* = 10) and cancer patients (*n* = 16). T cell recognition was identified using tetramer-staining analysis. Cytotoxicity assay was performed using as target cells TAP-deficient T2 cells loaded with MoA or the paired TuA.

**Results:**

A total of 66 unique pMHC recognized by CD8+ T cells across all groups were identified. Of these, 21 epitopes from microbiota were identified as novel immunological targets. Reactivity against selected TAAs was observed for both HS and CP. pMHC tetramer staining confirmed CD8+ T cell populations cross-reacting with CTA SSX2 and paired microbiota epitopes. Moreover, PBMCs activated with the MoA where shown to release IFNγ as well as to exert cytotoxic activity against cells presenting the paired TuA.

**Conclusions:**

Several predicted microbiota-derived MoAs are recognized by T cells in HS and CP. Reactivity against TAAs was observed also in HS, primed by the homologous bacterial antigens. CD8+ T cells cross-reacting with MAGE-A1 and paired microbiota epitopes were identified in three subjects. Therefore, the microbiota can elicit an extensive repertoire of natural memory T cells to TAAs, possibly able to control tumor growth (“natural anti-cancer vaccination”). In addition, non-self MoAs can be included in preventive/therapeutic off-the-shelf cancer vaccines with more potent anti-tumor efficacy than those based on TAAs.

**Supplementary Information:**

The online version contains supplementary material available at 10.1186/s13046-024-03004-z.

## Introduction

Approximately 10^14^ microbes are believed to be present in the human gastrointestinal tract. This corresponds to the number of cells and a DNA content 1,000 and 10,000 times greater than that in the human body, respectively [1]. The composition of the gut microbiota can vary during life owing to changes in diet, lifestyle, and habits. However, 90% of the species colonizing the gut microbiota belong to the *Firmicutes* and *Bacteroidetes* phyla [2, 3].

The bacteria that form the microbiota play a key role in human health. They are essential for intestinal digestion, prevention of pathogenic bacterial invasion, and regulation of the immune system [4, 5]. In addition to their physiological roles, the microbiota is actively involved in human diseases [6, 7], including tumor development and responses to treatments [8, 9]. Such a role has been mainly attributed to the production of specific metabolites, which may influence the genesis and development of cancer, as well as regulate the innate and adaptive immune responses [10–15].

A different perspective on the role of microbiota in cancer development and evolution is provided by the immunological mechanism based on the “molecular mimicry”. The latter is considered the major mechanism underlying immune disorders [16]. In particular, gut microbiota dysbiosis has been implicated in the activation of pathogenic T-cell responses, leading to gut-distal autoimmune diseases [17]. Activation of diabetogenic CD8^+^ T cells by molecular mimicry between microbial antigens of the gut microbiota and pancreatic islet autoantigens supports the evidence that cross-reactive CD8^+^ T cells can be elicited at the gut level with effects at distant sites [18]. Similarly, epitopes derived from microbiota (MoAs) may mimic tumor-associated antigens (TAAs) if they share identical or structurally similar amino acid residues at the same position along the epitope sequence. Therefore, the presentation of TAA-like MoAs to the immune system, in the context of MHC class I/II molecules, would elicit CD4^+^/CD8^+^ T cells cross-reacting with TAAs presented by tumor cells [19, 20].

Sporadic evidence for homology between MoAs and TAAs, together with T-cell cross-reactivity, has been previously reported [21–24]. Very recently, our group performed an unprecedented extensive analysis and found that sequence homology between TAAs and peptides from microbiota species of the Firmicutes and Bacteroidetes phyla is a frequent finding [25]. Most MoAs – TAAs paired epitopes share 6–7 identical residues or conservative substitutions along the sequence, with limited impact on the charge of the peptide. Strikingly, three of these pairs had identical sequences. Furthermore, the paired TAAs and MoAs are characterized by highly similar or even identical structural conformations, especially in the core TCR-facing residues with identical planar and dihedral angles. Finally, the areas of interaction with both HLA and TCR mostly match, suggesting that the paired peptides can be recognized by cross-reacting T-cells [25]. This may strongly influence the fate of tumor progression and provide a novel set of antigens for the development of next-generation anti-cancer therapeutic vaccines [26].

The present study shows that circulating CD8^+^ T cells that react with a large array of previously undescribed MoAs can be identified in both HS and CP. In addition, reactivity against TAAs was also observed in healthy individuals, suggesting previous priming by similar MoAs. Interestingly, CD8^+^ T cells cross-reacting with MAGE-A1 and paired MoAs were identified in three subjects.

## Materials and methods

### Peptide identification and epitope prediction of TAAs

Tumor-associated antigen (TAAs) epitopes for HLA-A* 02:01 were obtained from the Cancer Antigenic Peptide Database (https://caped.icp.ucl.ac.be/Peptide/list). Using the NetMHCpan 4.1 algorithm (https://services.healthtech.dtu.dk/service.php?NetMHCpan-4.1), these sequences were analyzed to identify the best nonamers with a predicted affinity value < 100 nM (Strong Binders, SB).

### BLAST homology search and MoAs epitopes prediction

The TAAs selected as SB according to the NetMHCpan 4.1 prediction tool were submitted to BLAST for a peptide alignment search against *Firmicutes* (taxid:1239) and *Bacteroidetes* (taxid:976) taxa within the non-redundant protein sequences database (https://blast.ncbi.nlm.nih.gov/Blast.cgi). For sequences with a higher level of similarity, a new prediction analysis was conducted with the NetMHCstabpan 1.0 (https://services.healthtech.dtu.dk/services/NetMHCstabpan-1.0/), and epitopes with a predicted affinity value < 100 nM and stability > 1 h were selected.

### Epitope modelling and molecular docking

The structural conformation of the predicted epitopes bound to HLA was evaluated by modifying the peptide included in the crystallized structure of HLA-A*02:01 deposited in the Protein Data Bank (https://www.rcsb.org). Briefly, the 1AO7 complex (PDB https://www.rcsb.org/structure/1AO7), which includes the HTLV-I LLFGYPVYV epitope crystallized with the HLA-A*02:01 molecule, and the α and β chains of TCR and β2-microglobulin were used as templates. The sequence of the peptide bound to MHC was modified and replaced with the selected nonamers using PyMol software (PyMol Molecular graphics system, version 1.8.6.2). The modified structure was then visualized using the Molsoft Mol Browser (version 3.8-7d).

### Samples collection

Peripheral blood was obtained from 15 cancer patients (5 hepatocellular carcinoma, 8 lung cancer, and 2 colon cancer with liver metastasis) and 10 healthy subjects. All samples were processed at the National Cancer Institute in Naples under informed consent, as approved by the Institutional Review Board. Fresh human peripheral blood mononuclear cells (PBMCs), isolated by density gradient centrifugation using Ficoll-Hypaque, were cryopreserved at −150 °C in FBS (Gibco, Thermo Fisher Scientific) plus 10% DMSO until analysis.

### DNA-barcoded pMHC-multimer library preparation

All peptides were synthesized with a purity of ≥ 90% (GenScript, Piscataway, NJ, USA). The lyophilized powders were reconstituted according to the manufacturer’s instructions.DNA barcoded multimer libraries for selected peptides were generated as previously described by Bentzen et al. [27]. Briefly, individual peptide–MHC (pMHC) complexes were generated by incubating for 1 h with 200 μM of each peptide and 100 μg/mL of HLA-A*02:01 MHC molecules using direct peptide loading [28]. The pMHC monomers were then coupled to a phycoerythrin (PE)- for TAAs peptides, or allophycocyanin (APC)- for MoAs-derived peptides, conjugated dextran backbone DNA barcode-labelled. Unique DNA-barcoded multimers were used to detect pMHC-specific T cells.

### Staining of antigen-specific T cells with DNA-barcoded pMHC multimers

PBMC from both cohorts were thawed and washed twice in RPMI1640 medium (Fischer Scientific 72400047) and 10% fetal bovine serum (FBS, Fischer Scientific 16140071). cells were then washed once in barcode cytometry buffer (BCB; PBS + 0.5% BSA + 100 mg/mL herring DNA + 2 mM EDTA) and incubated with DNA barcoded pMHC multimers for 15 min at 37 °C, followed by incubation at 4 °C for 30 min with CD8-BV480 (BD 566121) and dump channel antibodies CD4-FITC (BD 345768), CD14-FITC (BD 345784), CD19-FITC (BD 345776), CD40-FITC (Serotech MCA1590F), CD16-FITC (BD 335035), and a dead cell marker (LIVE/DEAD Fixable Near-IR, Invitrogen 2451278). The cells were washed twice with BCB, fixed in 1% paraformaldehyde (PFA), washed twice more, and resuspended in BCB. Cells were then acquired on a flow cytometer (AriaFusion, BD Biosciences); APC-pMHC multimer and double-positive PE/APC-pMHC multimer-binding CD8 + T cells were separately sorted (Suppl. Fig. 1). Sorted cells were centrifuged for 10 min at 5000 × g and the cell pellet stored at -20 °C.

### DNA-barcode sequence analysis

DNA barcodes from the isolated cells, as well as from an aliquot of the original multimer pool (10,000 × final dilution in the PCR reaction; used as a baseline) were amplified using a Taq PCR Master Mix kit (QIAGEN 201443) and 3 µL of forward and reverse primer (LGC Biosearch Technologies). Purified products (QIAquick PCR Purification Kit) were sequenced using PrimBio (PA, USA). DNA barcode sequencing data were processed using Barracoda software package2 (https://services.healthtech.dtu.dk/service.php?Barracoda-1.8). This tool identifies the DNA barcodes used in an experiment, assigns a sample ID and pMHC specificity to each barcode, calculates the number of reads and clonally reduced reads for each pMHC-associated DNA barcode, and includes statistical data processing. Fold change (FC) in read counts mapped to a given sample relative to the mean read counts mapped to triplicate baseline samples was estimated using normalization factors determined by the trimmed mean of M-values method. P-values were calculated by comparing each experiment individually to the mean baseline sample reads using a negative binomial distribution, with a fixed dispersion parameter set to 0.1. False discovery rates (FDRs) were estimated using the Benjamini–Hochberg method described by Bentzen et al. [27]. At least 1/1,000 reads associated with a given DNA barcode relative to the total number of DNA barcode reads in that given sample were set as the threshold to avoid false-positive detection of T cell responses. DNA barcodes with FDR < 0.1% (corresponding to *p* < 0.001) and Log2FC > 2 over the baseline values for the total pMHC library were considered significant and true T cell responses. The T cell frequency for each significantly enriched barcode was calculated from the percentage read count of the barcode relative to the percentage of CD8+ multimer+ T cells. A non-HLA-matching healthy donor sample was included as a negative control, and any T cell recognition determined in this sample was removed from the full dataset to exclude potential non-specific pMHC binding to T cells.

### T cell staining with pMHC tetramers

Specific matched peptides (TAA/microbiota) with a T cell response detected using DNA-barcode labelled multimers were selected to generate combinatorial fluorescently labelled pMHC tetramers [29, 30]. Single-fluorochrome pMHC tetramers were produced by conjugating individual pMHC complexes generated as described above to a library of fluorophore-labelled streptavidin (SA) molecules, including PE(Biolegend 405204), APC (Biolegend 405243), PE-CF594 (BD 562284), PECy7 (Biolegend 405206), BV421 (BD563259), and BV650 (BD 563855). pMHC molecules were incubated with their respective SA-conjugated fluorochromes for 30 min at 4 °C, followed by incubation with D-biotin (Sigma) (25 μM final concentration) for 20 min at 4 °C. pMHC tetramers for each specificity were generated in two colors and mixed at a 1:1 ratio before staining the cells.

PBMCs were thawed and washed with R10 + 10% fetal FCS. Cells were incubated with desatinib (50 nM final concentration) and 1 μL of pooled pMHC multimers per specificity for 15 min at 37 °C in 80 a total volume. cells were then mixed with 20 μL antibody staining solution containing CD8-BV480 (BD B566121) (final dilution 1/50), dump channel antibodies (CD4-FITC (BD 345768; final dilution 1/80), CD14-FITC (BD 345784; final dilution 1/32), CD19-FITC (BD 345776; final dilution 1/16), CD40-FITC (Serotech MCA1590F; final dilution 1/40), CD16- FITC (BD 335035; final dilution 1/64)), and a dead cell marker (LIVE/DEAD Fixable Near-IR (Invitrogen L34976; final dilution 1/1000)) and incubated for 30 min at 4 °C. Cells were washed twice in FACS buffer (PBS + 2% FCS) and acquired on an LSRFortessa flow cytometer (BD Biosciences).

### In vitro pre-immunization

To confirm the presence of cross-reacting CD8+ Tcells and their increase after a re-stimulation, PBMCs were cultured in presence of SSX2-BACT2 and SSX2-BACT3 peptides. Cells were plated at a density of 2 × 10^6^cells/mL in 3 mL of complete medium in a 6 well plate and stimulated with peptides at a final concentration of 10 uM in presence of 10 U/mL of IL-2 (Sigma) and 25 µL/mL of ImmunoCult™ Human CD3/CD28 T Cell Activator (StemCell technologies). After 5 days, cells were harvested, centrifuged at 1200 rpm for 5 min and stained with single-fluorochrome pMHC tetramers, generated as described above, and incubated with desatinib (50 nM final concentration) and 1 μL of pooled pMHC multimers per specificity (SSX2-PE; BACT2/BACT3-FITC) for 15 min at 37 °C in 80 a total volume. Cells were then mixed with CD8 PE-Cy7 (Life Technologies) and CD3 superbright 436 (Invitrogen) and incubated for 30 min at 4 °C. Cells were washed twice in FACS buffer (PBS + 2% FCS) and acquired on an AttuneNxT flow cytometer (LifeTechnologies).

### Interferon-gamma detection

PBMCs from three healthy HLA-A02:01 positive subjects were cultured in RPMI 1640 (Gibco) supplemented with 2 mM L-Glut (HyClone), 10% human serum (Sigma-Aldrich), 100 IU/ml penicillin and 100 μg/ml streptomycin (Capricorn). Cells were maintained at 37 °C in a humidified incubator with 5% CO2. PBMCs were seeded at 2.5 × 10^6^ cells/ml in 3 ml in a 6 well plate and cultured in presence of IL-2 (Sigma) at a final concentration of 10 U/mL and 25 µL/mL of ImmunoCult™ Human CD3/CD28 T Cell Activator (StemCell technologies). Following 3 days incubation, the interferon-gamma (IFN-γ) production was evaluated through the IFN-γ Secretion Assay –Cell Enrichment and Detection Kit (Miltenyi Biotec). Briefly, cells were harvested, centrifuged and incubated 4 h at 37 °C with SSX2, SSX2-BACT2 and SSX-BACT3 peptides at a final concentration of 10 uM. Unstimulated and PHA stimulated PBMCs were used, respectively, as negative and positive controls. Subsequently, cells were washed and stained with IFN-γ Catch Reagent, incubated 45 min at 37 °C, centrifuged and labelled with IFN-γ Detection Antibody (PE), CD8 PE-Cy7 (Life Technologies) and CD3 super bright 436 (Invitrogen). After 15 min incubation on ice, cell were washed, resuspended in 500uL of cold buffer and analysed by flow cytometry (AttuneNxT-LifeTechnologies).

### Flow cytometry analysis

All flow cytometry data were analyzed using the FlowJo data analysis software (version 10.8.1; FlowJo LLC). For antigen-specific T cell identification using combinatorial pMHC tetramer staining, we gated on single, live, CD3^+^, CD8^+^ lymphocytes and selected cells positive in two tetramer colors and negative in the remaining colors. For the IFN-γ detection, cells were gated as single, CD3^+^, CD8^+^ lymphocytes and double positive to CD8^+^ and IFN-γ.

### Cytotoxicity assay

Cytotoxic T Lymphocytes (CTLs) were generated from HLA-A*02:01 normal donor peripheral blood mononuclear cells (PBMC). PBMCs from four healthy HLA-A02:01 positive subjects were cultured in RPMI 1640 (Gibco) supplemented with 2 mM L-Glut (HyClone), 10% human serum (Sigma-Aldrich), 100 IU/ml penicillin and 100 μg/ml streptomycin (Capricorn). Cells were seeded at 2 × 10^6^ cells/ml in 3 ml in a 6 well plate in presence of IL-2 (Sigma) at a final concentration of 10 U/mL and 25 µL/mL of ImmunoCult™ Human CD3/CD28 T Cell Activator (StemCell technologies). PBMCs were stimulated with 10ug of SSX2-BACT3 peptide each 3 days for 5 times, cells without peptide were used as baseline control.

For cytotoxicity assay, T2 cells (174 × CEM.T2 CRL-1992-ATCC) were loaded with SSX2; SSX2-BACT2 and SSX2-BACT3 peptides at a concentration of 50 uM, incubated O/N at 27 °C, 2 h at 37 °C and with 1X Brefeldin A for 1 h and co-cultured with stimulated PBMCs for 5 h in a Target: Effector (T:E) ratio of 1:5. Specific cytotoxic activity was evaluated with Cell-mediated Cytotoxicity Assay kit (Immunochemistry Technologies).

### Data processing and statistical analysis

T cell recognition data determined by DNA-barcoded pMHC multimer analysis and all peptides with negative enrichment were set to LogFC equal to zero. GraphPad Prism6 was used to generate box plots, and related statistical analysis was used to visualize the flow cytometry data. For statistical analysis, data were assumed to have a non-Gaussian distribution, and non-parametric tests were used.

## Results

### Selection of TAAs and MoAs

**Fig. 1:**
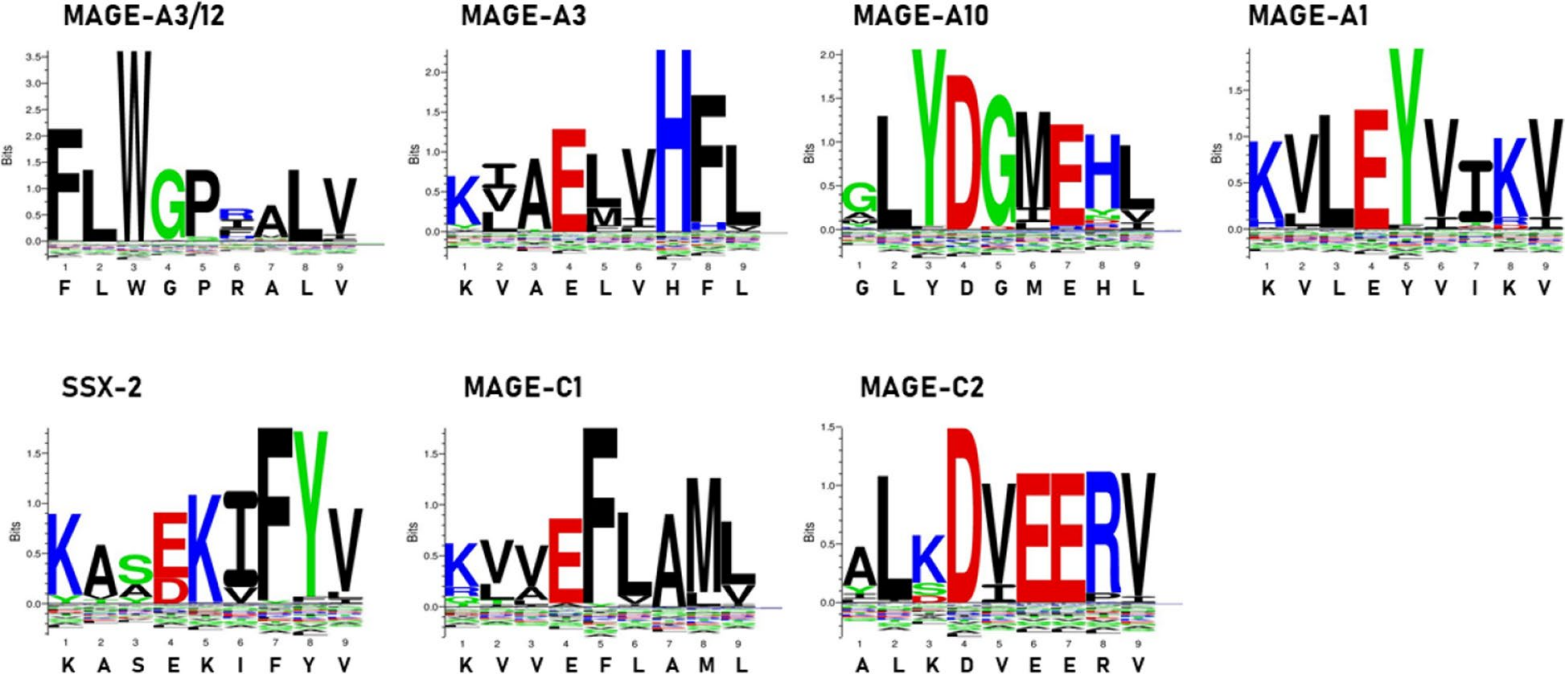
SeqLogo analysis of MoAs homologous to TAA: graphical representation of amino acids belonging to the consensus sequences of MoAs aligned to the corresponding homologous TAAs. Amino acid sequences from all the microbiota-derived epitopes with homology to each TAA were piled up to build sequence logos. The height of all aminoacids at each position indicates the sequence conservation at that position, while the height of each symbol within the stack indicates the relative frequency of each aminoacid at that position (https://services.healthtech.dtu.dk/service.php?Seq2Logo-2.0). Different colours indicate different classes of aminoacids

The paired TAAs and MoAs in the present study were derived from our previous analysis [25]. MAGE-A1, MAGE-A3, MAGE-A3/12, MAGE-A10, MAGE-C1, MAGE-C2, and SSX2 TAAs, together with 3–5 corresponding MoAs derived from *Firmicutes* and *Bacteroidetes* phyla were chosen (Table 1). MoAs were selected based on the predicted affinity to the same HLA allele as the corresponding TAA (HLA-A*02:01), with a maximum value lower than 100 nM. Indeed, peptides with such predicted values show 100% confirmation of HLA binding in an experimental setting based on TAP-deficient T2 cells [25, 31–34]. The overall mean of the predicted affinity values was 13.31 nM and 41 of the 53 peptides were below this value, suggesting very high binding affinity to the HLA-A*02:01 allele. The alignment of MoAs homologous to each TAA confirmed that, despite individual differences, the most predominant aa residues at each position always correspond to those in the TAA sequence (Fig. 1).

**Table 1:**
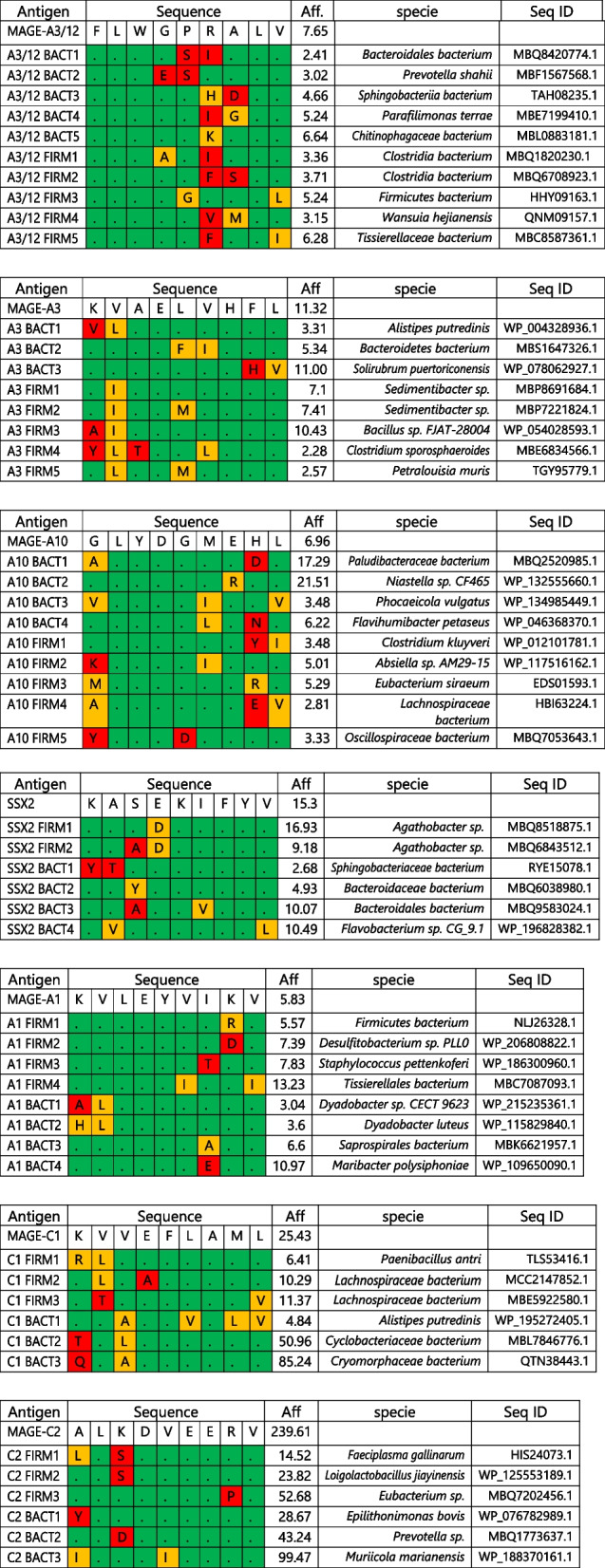
List of TAAs and paired MoAs selected for the study

The peptide sequence of each MoA is shown in relationship to the correlated TAA. Identical aa residues at a specific position along the sequence is shown as dot in green background. Different aa residues are shown in orange, if with the same chemical property, in red, if with different chemical property

### Conformation of selected TAAs and MoAs

**Fig. 2:**
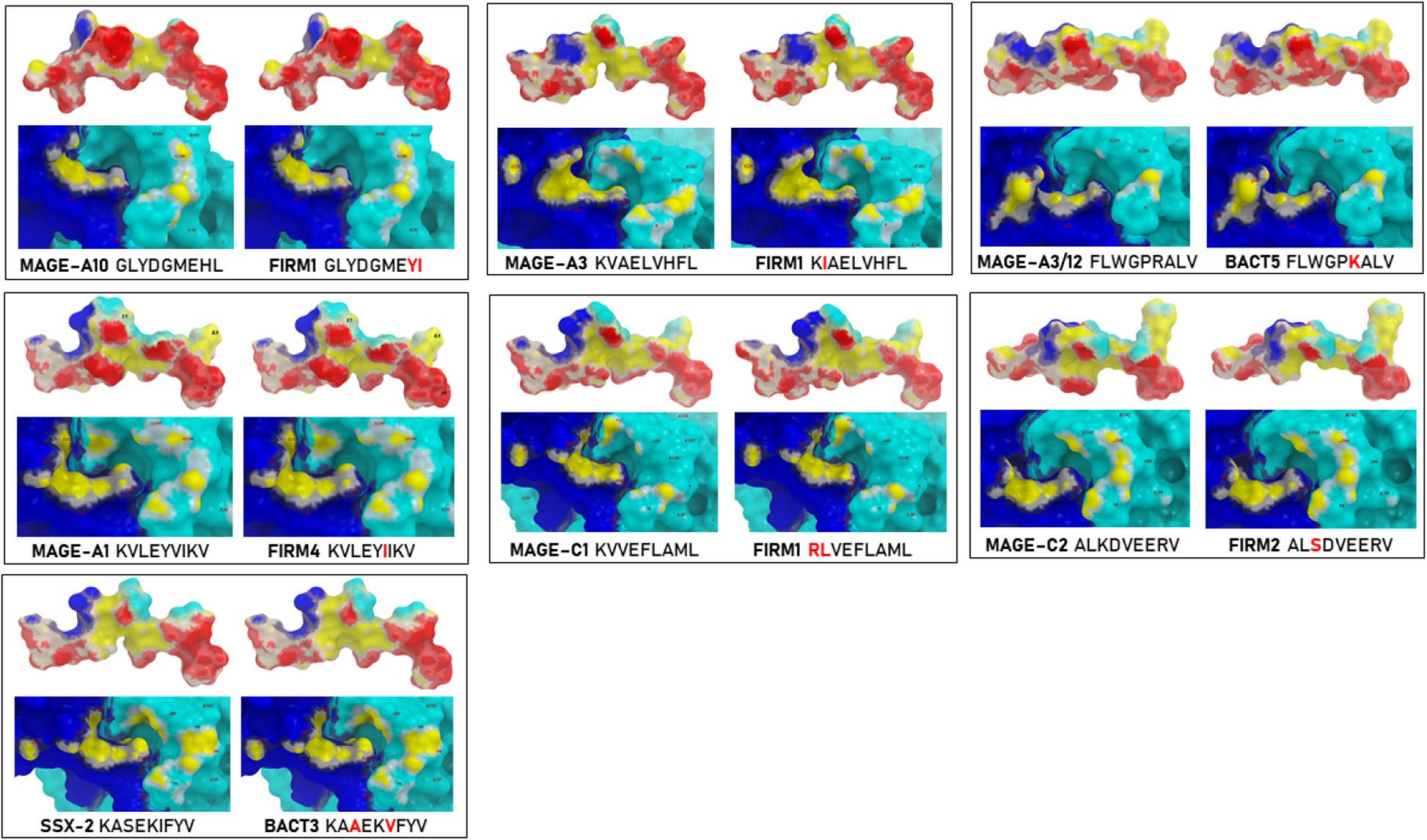
Predicted 3D conformation of TAA and microbiota-derived paired peptides and peptide-TCR interaction. The surface conformation of the most similar paired HLA-A*02:01 restricted TAA and MoAs-derived peptides is shown. Residues in the microbiota-derived epitopes (FIRM = firmicutes; BACT = bacteroidetes) that differs from the TAA sequences are indicated in red color. Red areas = contact points with HLA-A molecule; blue areas = contact points with TCR α chain; Light Blue = contact points with TCR β chain. The images below the peptides show the contact sites with α and β chains of TCR (yellow areas)

Epitope modelling and molecular docking were performed to prove that the sequence homology between the paired TAAs and MoAs was echoed in similar peptide conformations as well as contact areas with the HLA molecule and the TCR. The analysis confirmed that regardless of the position of the amino acid substitution along the peptide sequence, MoAs may have a similar, if not identical, conformation and pattern of contact with the α and β chains of the TCR, as shown by the footprints of the paired peptides (Fig. 2; Suppl. Figs. 2–15). The best examples of identical matching are the following: 1) the KVLEYVTKV peptide derived from *Staphylococcus pettenkoferi* with a Ile - Thr substitution at position 7 compared to MAGE-A1; 2) the KIAELVHFL peptide derived from *Sedimentibacter sp.* with a Val - Ile substitution at position 2 compared to MAGE-A3; 3) the FLWGPKALV peptide derived from *Chitinophagaceae bacterium* with a Pro - Lys substitution at position 6 compared to MAGE-A3/12; 4) the GLYDGMEYI peptide derived from *Clostridium kluyveri* with a His - Tyr and Leu - Ile substitution at position 8 and 9 compared to MAGE-A10; 5) the KTVEFLAMV peptide derived from *Lachnospiraceae bacterium* with a Val - Thr and Leu - Val substitution at position 2 and 9 compared to MAGE-C1; 6) the ALSDVEERV peptide derived from *Loigolactobacillus jiayinensis* with a Lys - Ser substitution at position 3 compared to MAGE-C2; and 7) the KVSEKIFYL peptide derived from *Flavobacterium sp. CG_9.1* with Ala - Val and Val-Leu substitutions at positions 2 and 9 compared to SSX2 (Fig. 2). In contrast, other MoAs presented substitutions that slightly or heavily affected the conformation as well as the pattern of contact with the α and β chains of the TCR (Suppl. Figs. 2–15).

### Epitope analysis

DNA-barcoded peptide-major histocompatibility complex (pMHC) multimers (HLA-A*02:01) were prepared with seven selected TAAs and 53 homologous MoAs from *Firmicutes* and *Bacteroidetes* phyla. In addition, a panel of 64 peptides derived from common viruses was constructed as an overall control of antiviral immune status. PBMCs from HLA-A*02:01 HS (*n* = 10) and CP (*n* = 15) patients were purified, incubated with DNA-barcoded pMHC multimers, and stained with a phenotype antibody panel to identify reactive CD8+ T cells [27].

**Fig. 3:**
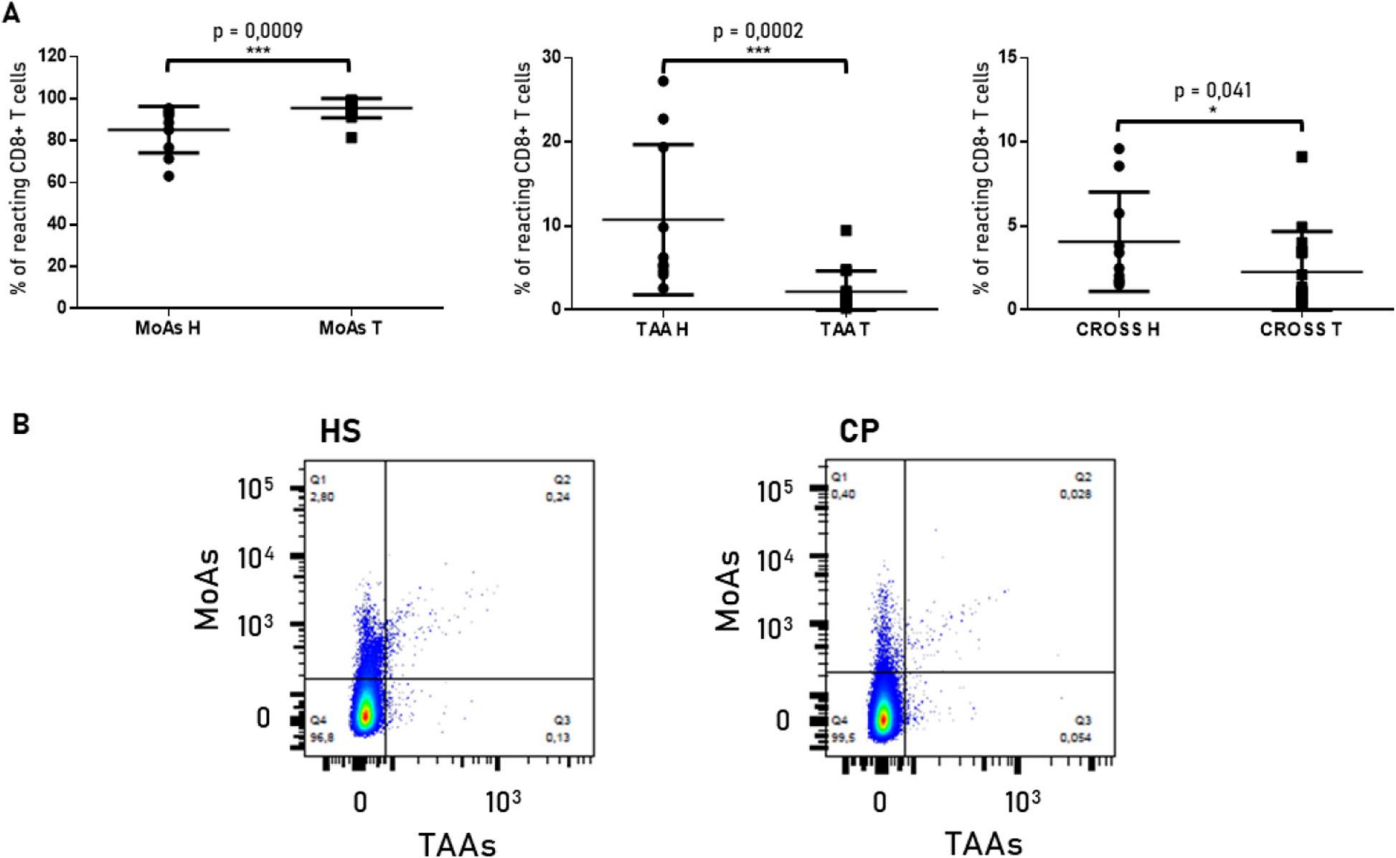
CD8^+^ T cells reacting with TAAs and MoAs. The plots (**A**) show the % of sorted CD8+ T cells reacting against the MoAs (bacteria + viruses), the TAAs or cross-reacting with TAAs and MoAs (CROSS) (*** = *p* < 0,0001, * = *p* < 0,005). **B** Representative box plot showing the % of sorted reactive CD8 + T cells for a healthy subject (left) and a cancer patient (right)

The percentage of CD8+ T cells reacting against MoAs was, on average, higher in CP (95.54%) than in HS (85.18%). In contrast, the percentage of CD8+ T cells reacting to TAAs or cross-reacting with TAAs and MoAs was, on average, higher in healthy individuals (10.75% and 4.07%, respectively) than in cancer patients (2.19% and 2.28%, respectively). This difference was statistically significant for all three comparisons (Fig. 3A). Strikingly, in both groups, three subjects showed a percentage of CD8+ T cells reacting against the TAAs and cross-reacting with TAAs and MoAs well above the average value (Fig. 3A, B).

### MoAs pMHC-DNA barcoding evaluation

**Fig. 4:**
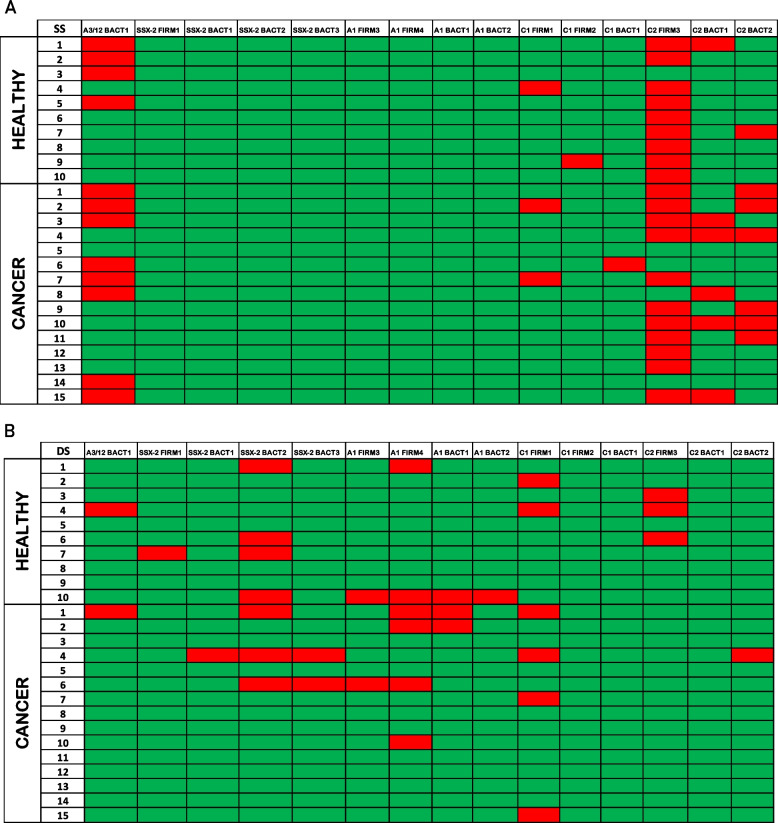
Reactivity to DNA-barcoded pMHC multimer MoAs. The statistically relevant (Log2FC > 2) reactivity to each peptide (column) from each subject (row) is enlighted in red. The different plots show reactivity against MoAs from the single positive (SS) (**A**) and from the double positive (DS) (**B**) sorted cell fraction

DNA barcodes with FDR < 0.1% (corresponding to *p* < 0.001) and Log_2_FC > 2 over the baseline values for the pMHC library were considered true and significant T-cell responses. The fraction of MoA-reacting T cells showed consistent binding to peptides homologous to MAGE-C2 (C2-BACT1, C2-BACT2, and C2-FIRM3) AND MAGE-A3/12 (A3/12-BACT1) in both tumor patients and HS. Scattered binding to peptides homologous to MAGE-C1 (C1-BACT1, C1-FIRM1, C1-FIRM2) was observed in both groups (Fig. 4A). Interestingly, while binding to such peptides was observed in scattered samples, a completely different predominant pattern was observed in the fraction of cross-reacting T cells. Indeed, the peptides that were more frequently bound were those homologous to MAGE A1 and SSX2 (A1-BACT1, A1-BACT2, A1-FIRM3, A1-FIRM4) (SSX2-BACT1, SSX2-BACT2, SSX2-BACT3, SSX2-FIRM1) with an equal distribution between the two groups (Fig. 4B). The number of peptides bound by cross-reactive T cells was broadly different in both groups, ranging from 0 to 5 in healthy individuals and from 0 to 7 in tumor patients (Suppl. Fig. 16). Binding of peptides homologous to other TAAs was not observed. Consistent binding to the positive control CMVpp65 peptide was observed in of the 21/25 subjects in both groups (data not shown).

### TAAs pMHC-DNA barcoding evaluation

**Fig. 5:**
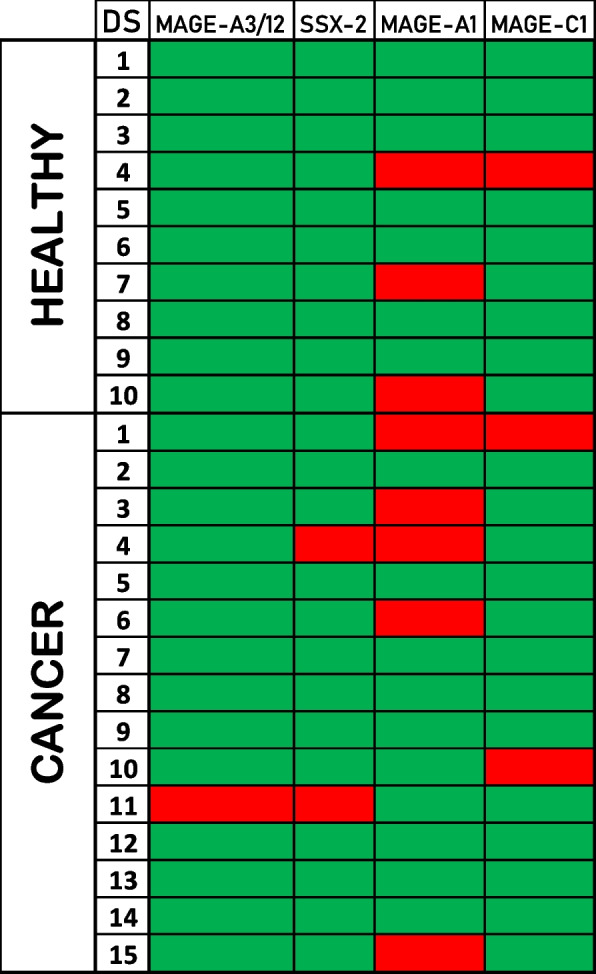
Reactivity to DNA-barcoded pMHC multimer TAAs. The statistically relevant (Log2FC > 2) reactivity to TAAs from each subject (row) is enlighted in red. The table shows the reactivity to TAA epitopes in both HS and CP

The analysis of the fraction of double-positive T cells revealed binding to TAAs in both HS and CP, and most of this binding was specific to MAGE-A1. Three (3) HS (H-004, H-007, and H-010) showed binding to the TAAs. Binding values to MAGE-A1 peptide by H-007 and H-010 samples showed a > 2log fold increase, with a high statistical significance (*p* < 1 × 10^–6^) (Fig. 5A). In addition, binding values to MAGE-A1 and MAGE-C1 peptides by H-004 sample showed a statistical significance (*p* < 0.005) with a fold increase nearly reaching the 2log fold increase. Seven (7) CP showed binding to TAAs (T-001, T-003, T-004, T-006, T-010, T-011, and T-015), and some of them to more than a single TAA. Binding values to MAGE-A1 peptide by T-001, T-003, T-006, and T-015 samples showed a > 2log fold increase, with a high statistical significance (*p* < 1 × 10^–6^), while T-004 and T-011 samples showed a binding value with a statistical significance (*p* < 0.005) and a fold increase nearly reaching the 2log fold increase. Binding values to MAGE-C1 peptide by T-001 and T-010 samples, to SSX2 peptide by T-004 and T-011 samples, and to MAGE-A3/12 peptide by T-011 sample showed a > 2log fold increase, with a statistically significant difference (*p* < 1 × 10^–6^). In addition, the T-004 sample showed a binding value to A3/12 peptide with statistical significance (*p* < 0.005) and a fold increase nearly reaching the 2log fold increase. Overall, the samples that reacted with more than single peptides were T-001, which bound MAGE-A1 and MAGE-C1 peptides; T-004, which bound MAGE-A1, SSX2, and MAGE-A3/12 peptides; and T-011, which bound MAGE-A1, SSX2, and MAGE-A3/12 peptides (Fig. 5).

### TAAs and MoAs cross-reactivity

**Fig. 6:**
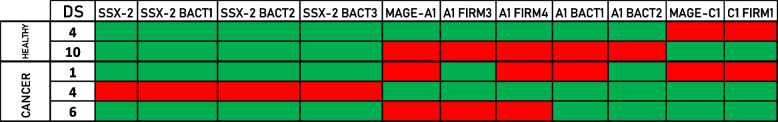
Double reactivity to DNA-barcoded pMHC multimer TAAs/MoAs. The statistically relevant (Log2FC > 2) reactivity to TAAs and MoAs from each subject (row) is enlighted in red. The table shows the reactivity to homologous coupled peptides (TAA and MoAs) in both HS and CP

According to the pMHC-DNA barcoding evaluation, two samples from the HS and three samples from the tumor patients showed double positivity for binding to homologous TAAs and MoAs with a > 2log fold increase and a highly statistical significance (*p* < 1 × 10^–6^). In particular, the H-010, T-001, and T-006 samples showed binding to MAGE-A1 and, all three, the homologous A1-FIRM4, and H-010 bound the A1-FIRM3 (T-006), A1-BACT1 ( T-001), and A1-BACT2 peptides. H-004 and T-001 samples bound to MAGE-C1 and homologous C1-FIRM1. Finally, the T-004 sample bound to SSX2 and the homologous SSX2-BACT1, SSX2-BACT2, and SSX2-BACT3 peptides (Fig. 6). In all such double reactivities, the paired TAAs and MoAs show highly similar, if not identical, conformation and contact areas to both HLA and TCR α and β chains (Fig. 2 and Suppl. Figs. 2–15).

###  Validation of CD8^+^ T cell cross-reactivity

To confirm that the double positivity corresponded to true T cell cross-reactivity to the paired TAAs and MoAs, tetramer-staining analyses were performed.

Because of the limited availability of stored samples, the analysis was performed on sample T-004 only, which showed a broad reactivity against the SSX2 TAA and SSX2-BACT1, BACT2, and BACT3 MoAs during the DNA-barcoding screening.

**Fig. 7:**
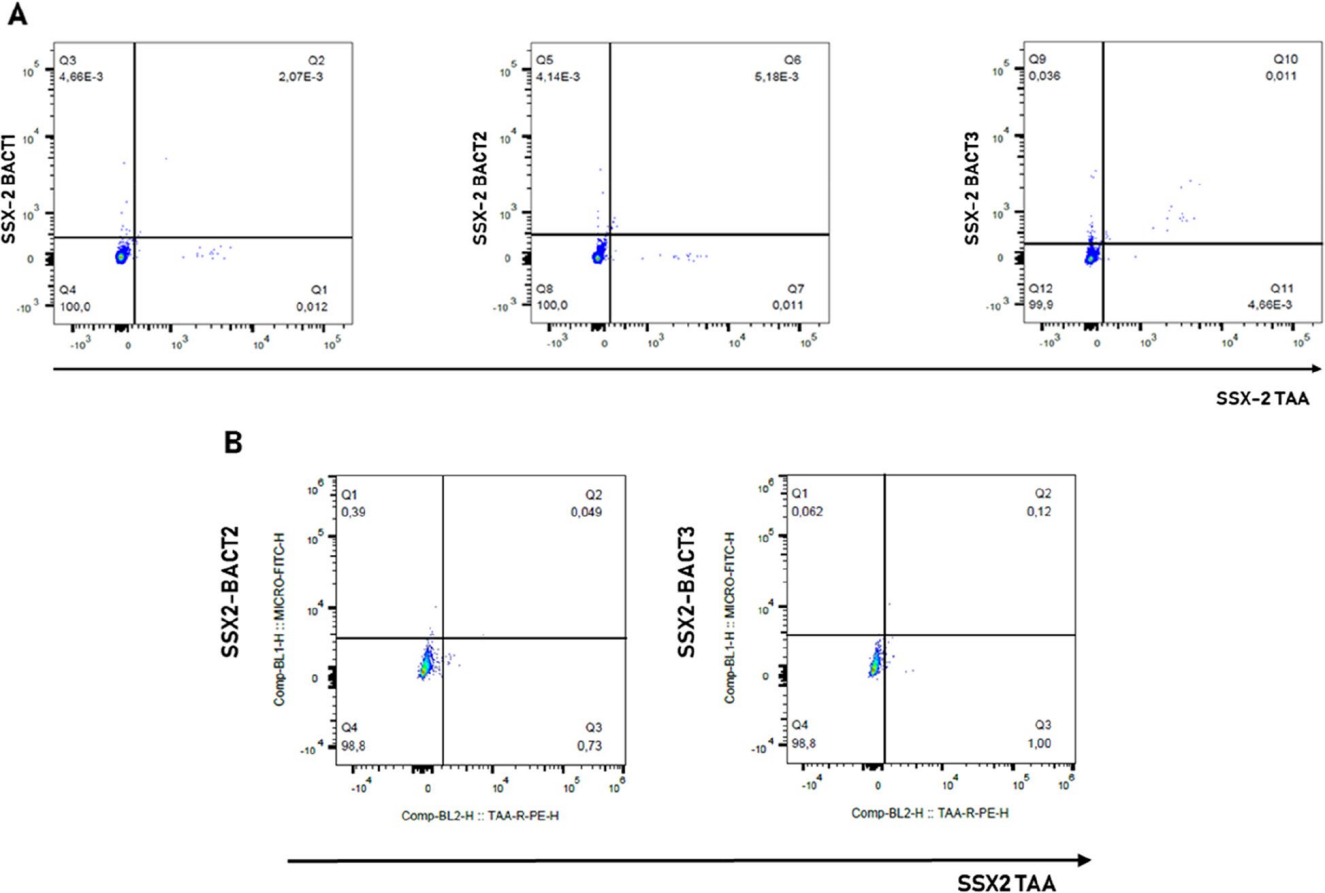
**A** Cross-reactive CD8 + T cells in pMHC tetramer staining. Unstimulated PBMCs from sample T-004 were incubated with pMHC tetramers loaded with TAA and MoAs homologous peptides. Dot plots show the reactivity against the SSX2 peptide (PE-A) and SSX2-BACT1 (APC-A), SSX2-BACT2 (PE-CF594-A) and SSX2-BACT3 (PE-Cy7-A). **B** Cross-reactive CD8^+^ T cells in pMHC tetramer staining after in vitro pre-immunization. Cross-reactivity against the paired SSX2-BACT2/BACT3 peptides was evaluated after an in vitro pre-immunization. PBMCs were stimulated with MoAs derived epitopes and tetramer staining was used to assess the cross-recognition of homologous TAA peptide

Tetramer staining showed the cross-reactivity of CD8^+^ T cells with the SSX2 peptide and each of the paired MoA. Unstimulated PBMC were analyzed by flow cytometry using paired fluorescent HLA-A2/peptide tetramers and CD8-specific Abs. SSX2-, MoA-, and cross-reacting T cells were detected in the unstimulated PBMC of T-004 tumor patients. The SSX2-reacting CD8^+^ T cells were approximately 0.01%, and the MoA-reacting CD8^+^ T cells were approximately 0.004% for BACT1 and BACT2 and 0.036% for BACT3 pepide. Different levels of cross-reacting CD8^+^ T cells were observed in all three comparisons. The percentage of such CD8+ T cells was directly correlated with that observed for single peptide reactivity. The highest percentage of cross-reacting CD8^+^ T cells (0.011%) was observed in the SSX2/BACT3 comparison (Fig. 7A). The presence of circulating primed T cells specific for the MoAs, homologous to TAAs, was assessed by an ex vivo immunization of PBMCs. Isolated PBMCs were stimulated with SSX2-BACT2 and SSX-BACT3 peptides for 5 days and CD8^+^ Tcell cross recognition of both MoAs and homologous SSX2 derived epitopes was assessed via tetramer staining. The results showed an increase in T cell recognition of both BACT2/3 and SSX2 epitopes, together with an increasing frequency of cross-reacting CD8+ Tcells as showed in Fig. 7B.

### IFN-γ release after peptide stimulation

The activation of antigen-specific CD8^+^ Tcells was evaluated by the production of IFN-γ after stimulation with SSX2, SSX2-BACT2 and SSX2-BACT3 peptides.

**Fig. 8:**
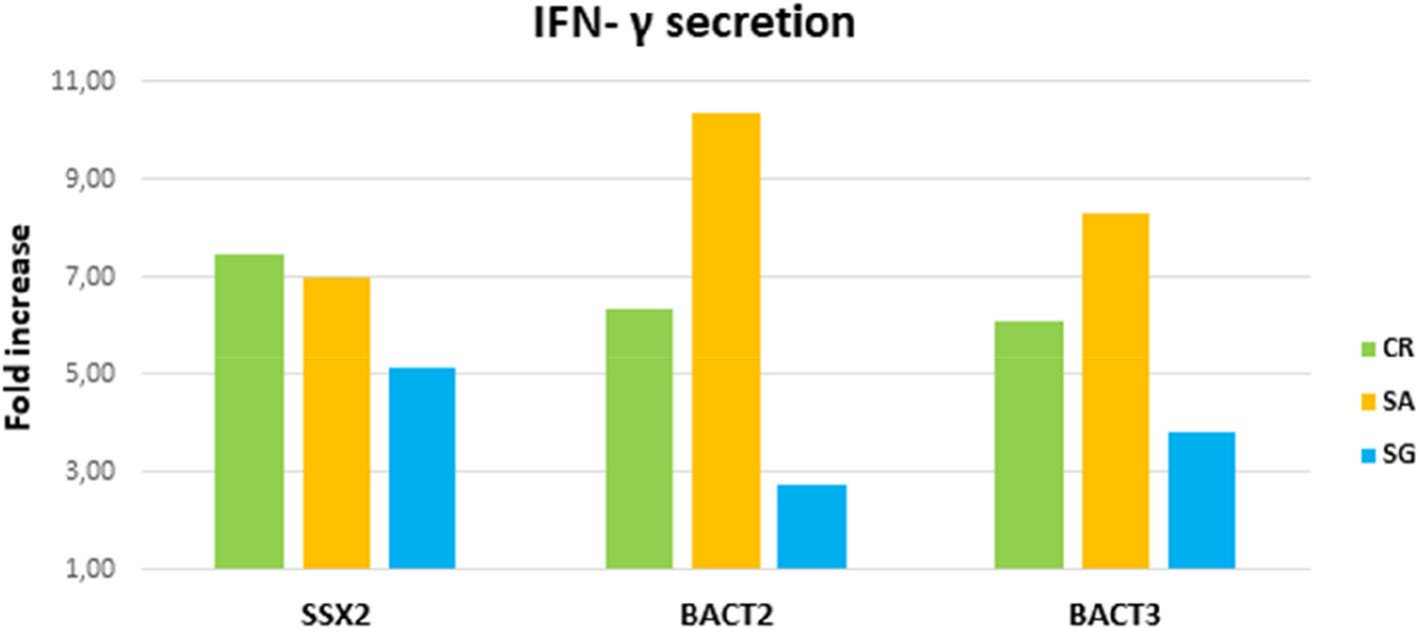
IFN-γ release by CD8^+^ T cells. Secreted IFN-γ was detected using the secretion assay on viable IFN-γ^+^ CD8^+^ Tcells. The graph shows the increase in the IFN- γ production after the peptide stimulation

The flow cytometric analysis of IFN-γ release revealed a relevant production in all subjects (Fig. 8), with an average fold increase against the non stimulated controls of 6.52 for SSX2, 6.48 for SSX2-BACT2 and 6.07 for SSX2-BACT3.

### Cross-reactive CTL activity

The final proof of cross-reactivity was provided by assessing the cytotoxic activity of PBMCs stimulated ex vivo with the MoA-derived peptide BACT3 on TAP-deficient T2 cells loaded with SSX2, SSX2-BACT2 or SSX2-BACT3 peptides.

**Fig. 9:**
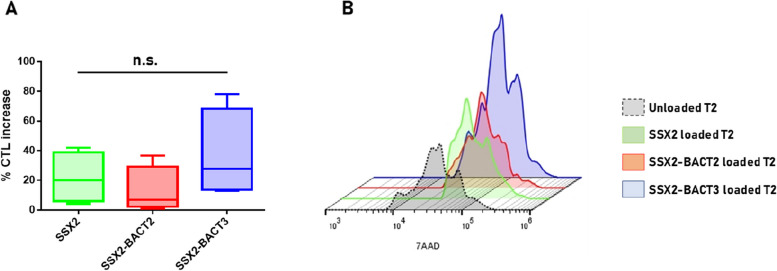
CTL activity. Cytotoxic activity of PBMCs was assessed by fluorimetric assay in TAP-deficient T2 cells loaded with the indicated peptides. **A** Average percentage increase of CTL activity over T2 control cells; **B** example of layout result in a single individual, showing increase in 7AAD fluorescence intensity

The results showed a cross-reactive killing activity of activated PBMCs on T2 cells presenting each of the three peptides. Interestingly, the average percentage increase of CTL activity did not reach the statistical difference in the three settings, suggesting the comparable efficient targeting of T2 cells presenting one of the paired antigens (Fig. 9).

## Discussion

A high number of microorganism-derived antigens (MoAs) showing sequence and conformational homology with tumor-associated antigens (TAAs) have been recently reported, and their implication in eliciting cross-reacting anti-cancer T cells has been proposed [25, 35, 36]. In the present study, we aimed to confirm that MoAs predicted from extracellular bacteria that form the microbiota are recognized by CD8^+^ T cells. Consequently, the presentation of such MoAs in the context of MHC class I molecules is reasonable. Moreover, the cross-reactivity of CD8+ T cells against MoAs and homologous TAAs was investigated in patients with HS and tumors.

The selection of homologous MoAs and TAAs was based on previous observations by our group, and for each TAA, 3–5 corresponding MoAs derived from *Firmicutes* and *Bacteroidetes* phyla were chosen [25]. All selected TAAs belong to the cancer testis (CT) subgroup. In particular, antigens belonging to the Melanoma Antigen Gene family (MAGE-A1, MAGE-A3, MAGE-A3/12, MAGE-A10, MAGE-C1, MAGE-C2) and SSX2 are found to be broadly expressed in many tumor types [37, 38].

When compared to the corresponding TAA, each MoA showed a similar, if not higher, predicted affinity to the HLA molecule, despite 1–2 amino acid differences. Nevertheless, the consensus sequence derived from all selected MoAs was identical to the corresponding TAA. Conformation analyses revealed highly overlapping structures between homologous TAAs and MoAs, with indistinguishable contact areas with both HLA molecules and TCR α and β chains. Overall, this strongly suggests the induction of CD8^+^ T cells cross-reacting with TAAs and MoAs. A few exceptions to this general observation have been found, especially in the residues interacting with TCR α and β chains, when the substituting amino acid residue in the MoA was of a different chemical/structural group.

T cell binding screening was based on a panel of DNA-barcoded peptide-major histocompatibility complex (pMHC) multimers (HLA-A*02:01), including all seven selected TAAs and 53 homologous MoAs, together with 64 peptides derived from common viruses. Unstimulated CD8^+^ T cells from subjects in both experimental groups showed a high level of reactivity to MoAs, which was significantly higher in CP than in HS (95.54% vs. 85.18%). In contrast, reactivity to TAAs and cross-reactivity to MoAs and TAAs were significantly higher in HS than in CP (10.75% vs. 2.19%; 4.07% vs. 2.28%, respectively). Such unexpected observations could be reasonably explained by the priming of HS by MoAs, eliciting a T cell response that cross-reacts with the corresponding TAAs.

The evaluation of specific MoAs bound by CD8^+^ T cells showed unique patterns when sorted by single staining (T cells binding only MoAs) or double staining (T cells binding MoAs and TAAs). Single-stained (SS) T cells were found to bind MoAs homologous to MAGE-C2 and MAGE-A3/12 TAAs, in particular C2-FIRM3 (20/25 samples) and A3/12-BACT1 (12/25). Double-stained (DS) T cells, instead, were found to bind MoAs homologous to MAGE-A1 and SSX2 TAAs, in particular A1-FIRM4 (6/25 samples) and SSX2-BACT2 (7/25). The C2-FIRM3 ALKDVEEPV peptide is derived from the AMP-binding proteins of Eubacterium sp. and Clostridia bacterium. The A3/12-BACT1 FLWGSIALV peptide is derived from the cation-translocating P-type ATPase of the Bacteroidales genus. The A1-FIRM4 KVLEYIIKI peptide is derived from the ATP-binding protein of the genus Tissierellales. Finally, the SSX2-BACT2 KAYEKIFYV peptide was derived from an alpha/beta hydrolase of the Bacteroidaceae genus. None of these peptides were present in the Immune Epitope Database and Analysis Resource (iedb.org), representing the newly identified microbiota-derived MHC class I-associated epitopes. The striking consistent T cell reactivity for the C2-FIRM3 and A3/12-BACT1 peptides is likely explained by the presence of Eubacterium sp. and Bacteroidales bacterium in the universal microbiota phylogenetic core, independent of lifestyle and country of origin [39]. In particular, the Eubacterium spp. populations in the gut has been shown to be positively correlated with the Mediterranean diet [40].

Double-stained (DS) T cells were found to bind essentially MAGE-A1 TAA (8/25), and three subjects (H-010, T-001, and T-006) showed T cells binding both MAGE-A1 and the corresponding MoAs. Such a result may have a significant impact on a large spectrum of cancer subtypes. Indeed, MAGE-A1 is overexpressed in a significant percentage (≥ 20%, on average) of different tumor types, including colon [41], melanoma [42], and lung [43], as well as in a low percentage (~ 10%) of breast [44] and liver cancers [45]. Similarly, the anecdotal observed T-cell cross-reactivity against SSX2 (T-004) or MAGE-C1 (H-004) and their homologous MoAs may be highly relevant. Indeed, SSX2 and MAGE-C1 are overexpressed in various cancers [39, 46]. We further showed that circulating T cells primed by MoAs were recalled and expanded by an in vitro immunization protocol. Such T cells reacted against TAA in a tetramer-staining analysis producing a relevant increased levels of IFN-γ. Furthermore, PBMCs ex vivo activated with the SSX2-BACT3 peptide showed a comparable cytotoxic activity against TAP-deficient T2 cells loaded with either the same peptide or the homologous SSX2 TAA or SSX2-BACT2 peptides. These results provided the conclusive proof that, indeed, T cells activated by a MoA cross-react with an homologous TAA, exerting a cytotoxic killing activity on target cells expressing the TAA.

Overall, T cell cross-reactivity against TAAs elicited by homologous MoAs may represent a potent immunological shield against a broad spectrum of cancers that can prevent tumor growth in healthy subjects or improve clinical prognosis in cancer patients. To this end, it is unfortunate that the three CP showing cross-reactive T cells in the present study were lost to follow-up, and information about clinical progression was not available.

The functional analysis in a preclinical model will definitely demonstrate the anti-tumor effect of the described cross-reactive T cells.

In conclusion, the data described provide the first large report of several MoAs, some of which have not been reported before, homologous to TAAs recognized by T cells, and cross-reactivity was observed in both HS and CP. Further studies on larger numbers of HS and CP patients will provide validation with a high potential impact on cancer immunotherapy. Indeed, non-self MoAs would become a key tool for developing preventive/therapeutic “multi-cancer” vaccine strategies with much stronger immunogenicity compared to the corresponding self-TAAs.

### Supplementary Information

**Additional file 1: Suppl. Fig. S1.** Descriptive flow cytometry plots for gating strategy on healthy donors and cancer patients’ PBMCs stained with DNA-barcoded pMHC multimers and surface antibody markers to sort viral/microbiome-derived (APC) and TAA/double positive (PE/PE+APC) multimer+ CD8+ T cells and to quantify multimer+ CD8+ T cells. **Suppl. Figs. S2-15.** Predicted 3D conformations of TAA and microbiota-derived paired peptides. The surface conformation of the paired TAA MoA-derived peptides is shown. Residues in the Mo epitopes that differed from the TAA sequences are indicated in red. A-Red areas  = contact points with HLA-A molecule; blue areas = contact points with TCR α chain; Light Blue  areas = contact points with TCR β chain. The images below the peptides show contact sites with the TCR (yellow areas). **Suppl. Fig. S16.** Diagrams show the total number of significant responses in DNA-barcoded pMHC multimers for HS (A) and CP (B).

## Data Availability

Data and material have been deposited and are publicly available at 10.5281/zenodo.10817340.
