# Supplementary material for: Cross-reactive CD8+ T cell responses to tumor-associated antigens (TAAs) and homologous microbiota-derived antigens (MoAs)
Source: J Exp Clin Cancer Res. 2024 Mar 20;43:87. doi: 10.1186/s13046-024-03004-z (PMC10953141; doi:10.1186/s13046-024-03004-z)

Suppl. Fig. S1

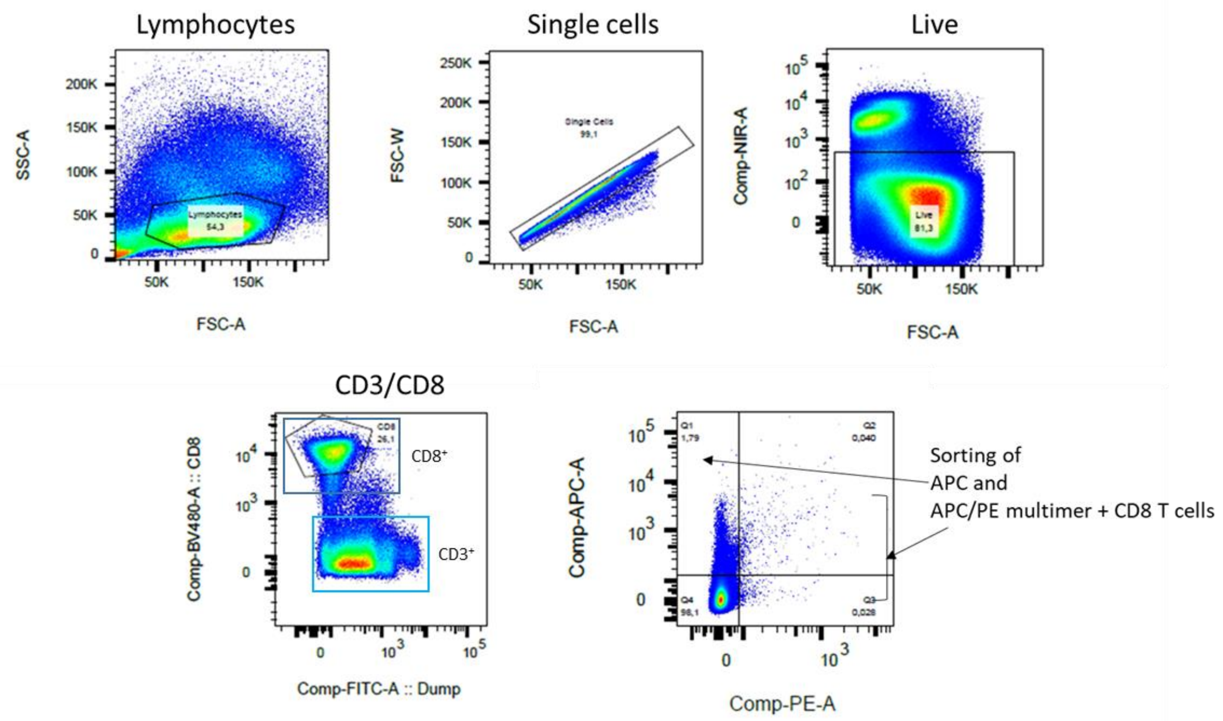
Lymphocytes
Single cells
Live
CD3/CD8
SSC-A
FSC-A
FSC-W
Comp-NIR-A
Comp-BV480-A :: CD8
Comp-FITC-A :: Dump
Comp-APC-A
Comp-PE-A
CD8⁺
CD3⁺
Sorting of
APC and
APC/PE multimer + CD8 T cells

MAGE-A1

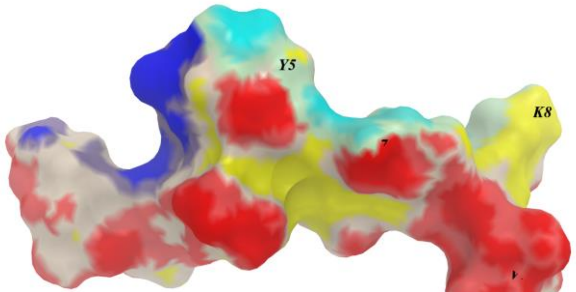

KVLEYVIKV

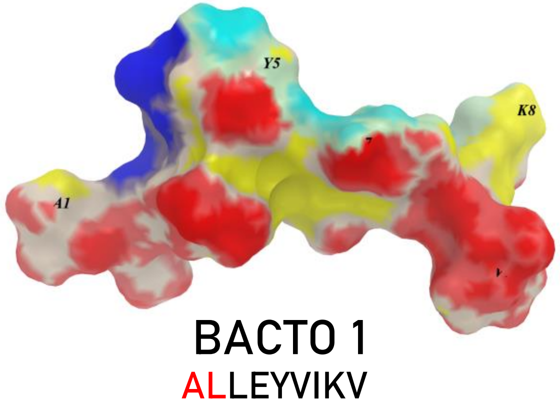

BACTO 1
ALLEYVIKV

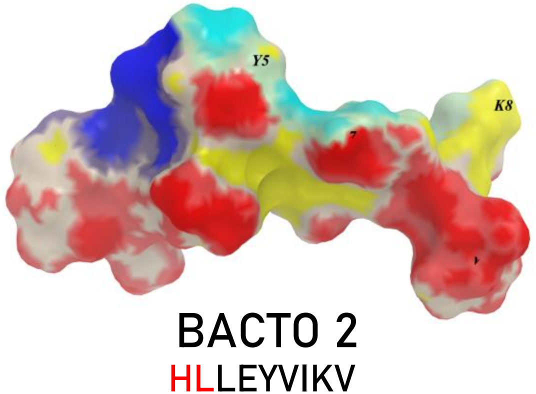

BACTO 2
HLLEYVIKV

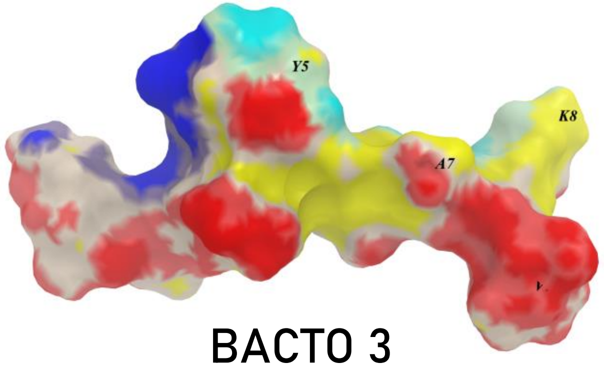

BACTO 3
KVLEYVAKV

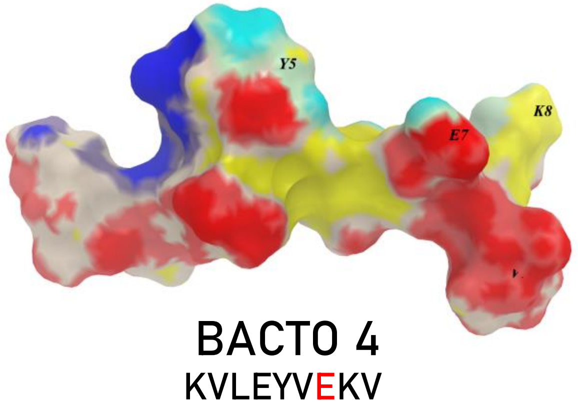

BACTO 4
KVLEYVEKV

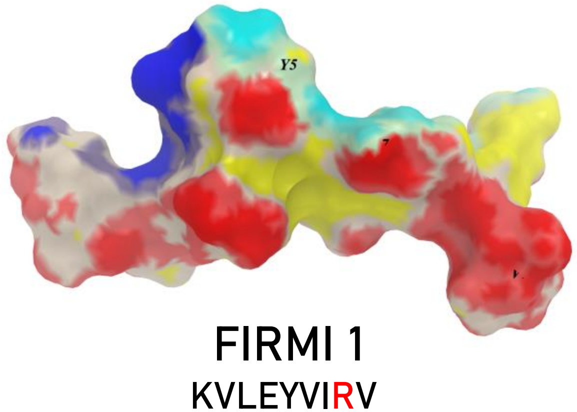

FIRMI 1
KVLEYVIRV

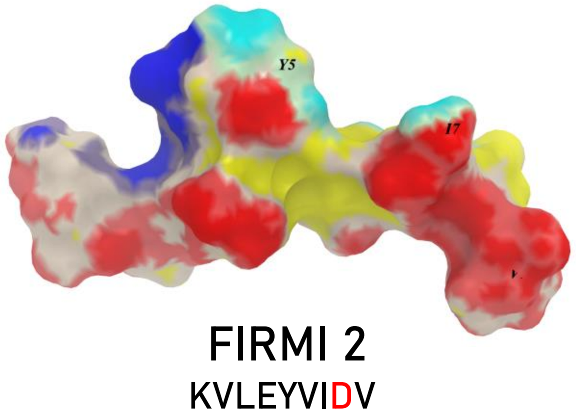

FIRMI 2
KVLEYVIDV

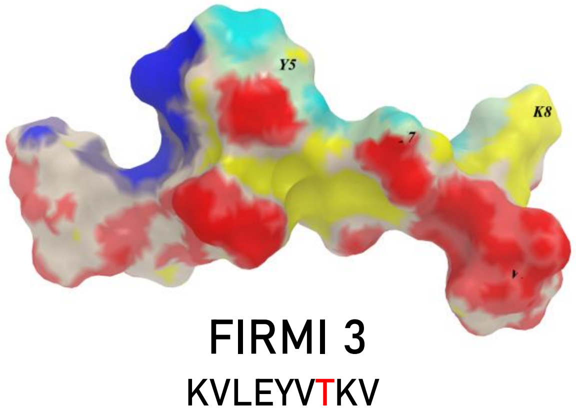

FIRMI 3
KVLEYVTKV

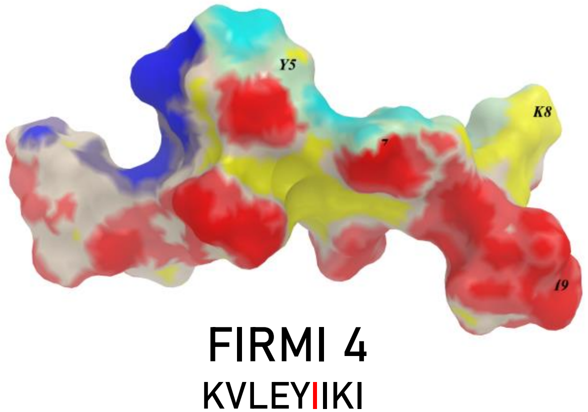

FIRMI 4
KVLEYIIKI

Suppl. Fig S3

MAGE-A1

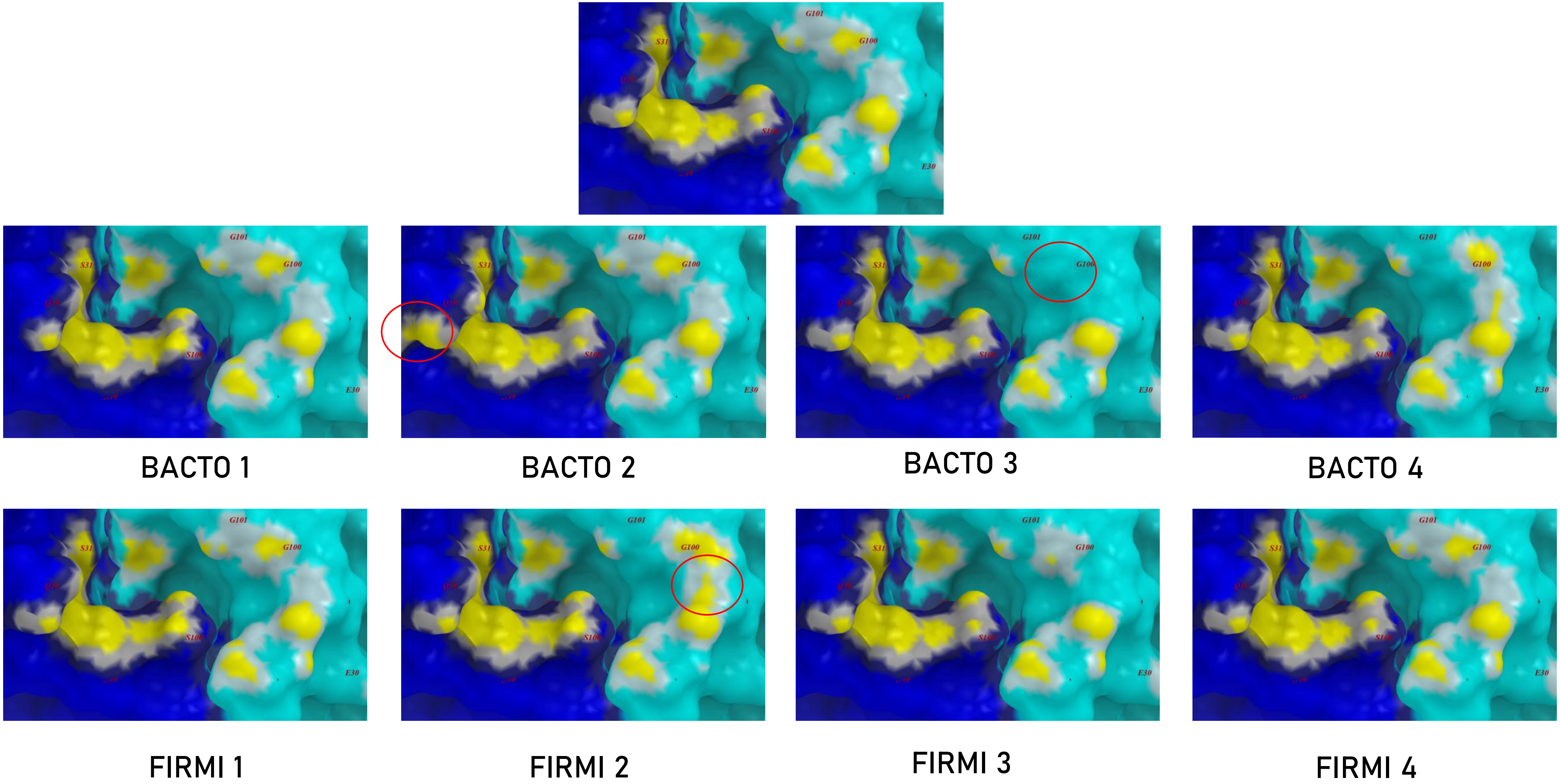

Suppl. Fig S4

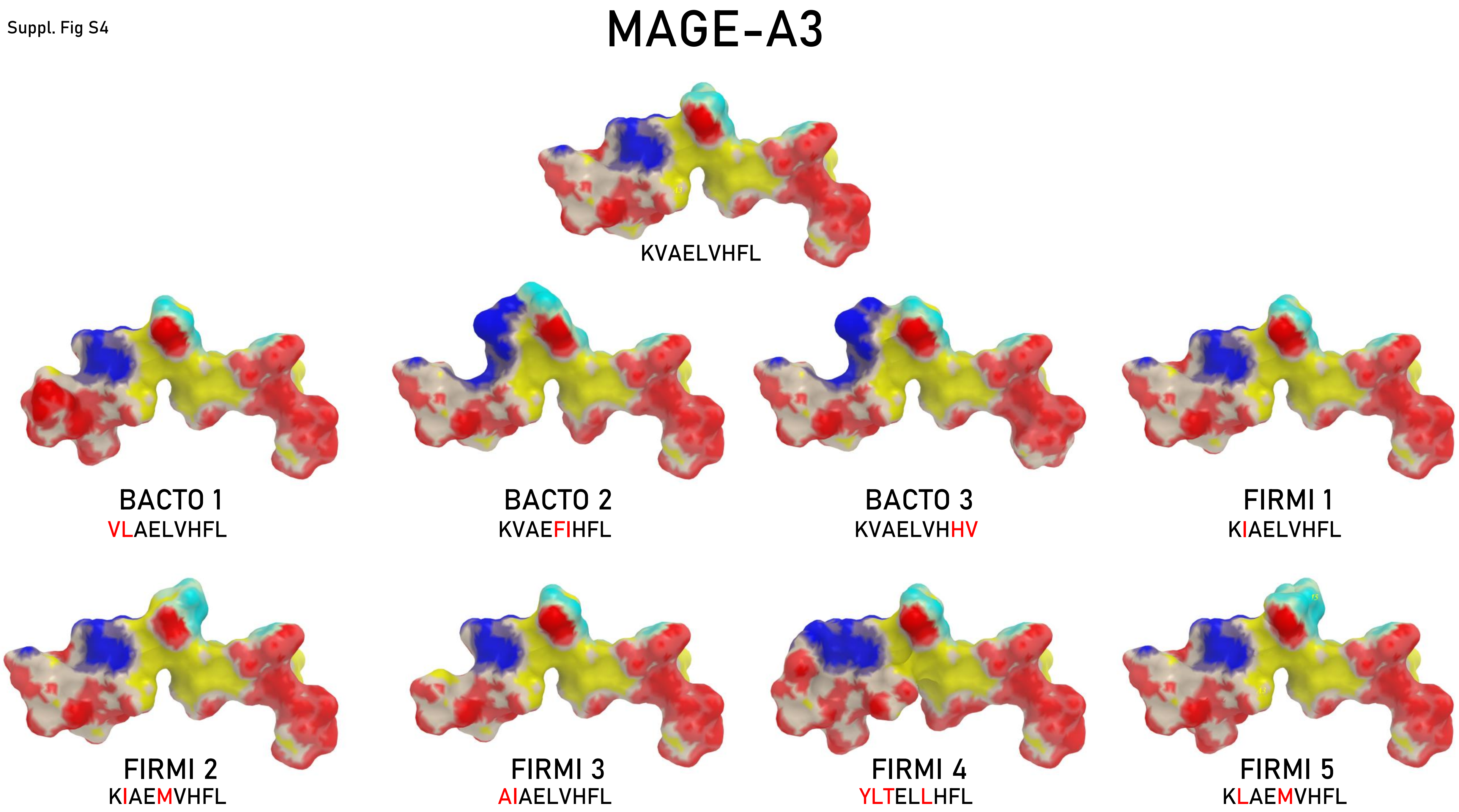
MAGE-A3
KVAELVHFL
BACTO 1
VLAELVHFL
BACTO 2
KVAEFIHFL
BACTO 3
KVAELVHHV
FIRMI 1
KIAELVHFL
FIRMI 2
KIAEMVHFL
FIRMI 3
AIAELVHFL
FIRMI 4
YLTELLLHFL
FIRMI 5
KLAEMVHFL

Suppl. Fig S5

MAGE-A3

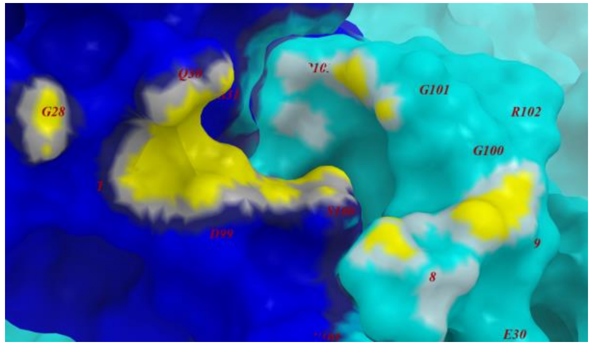

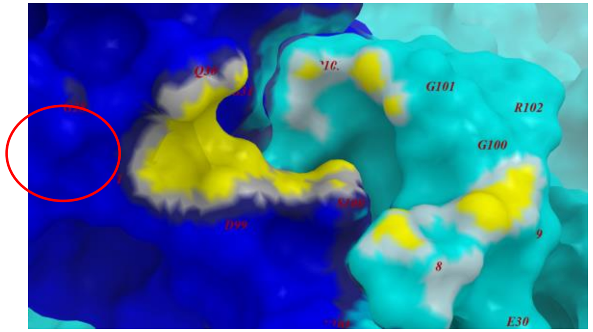

BACTO 1

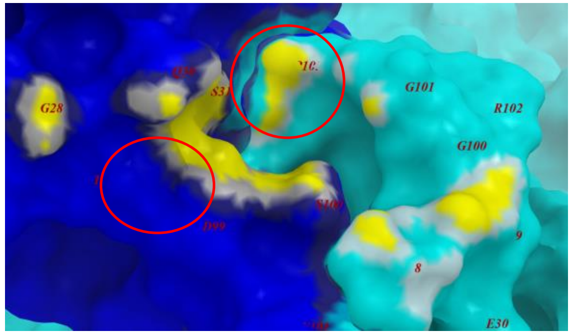

BACTO 2

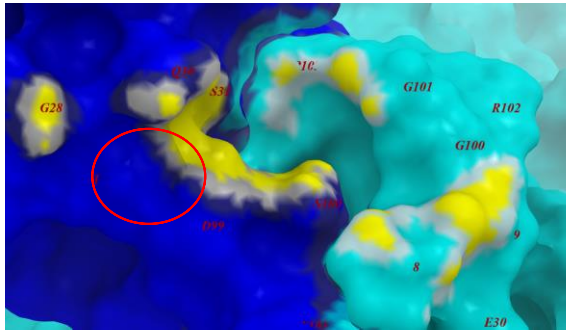

BACTO 3

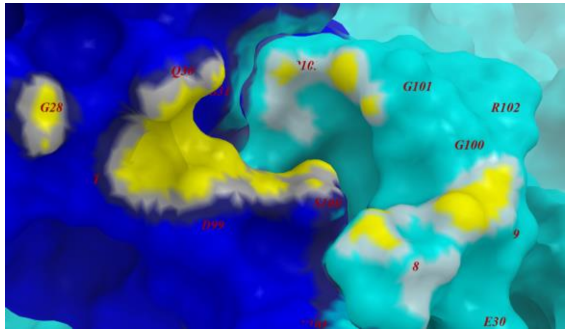

FIRMI 1

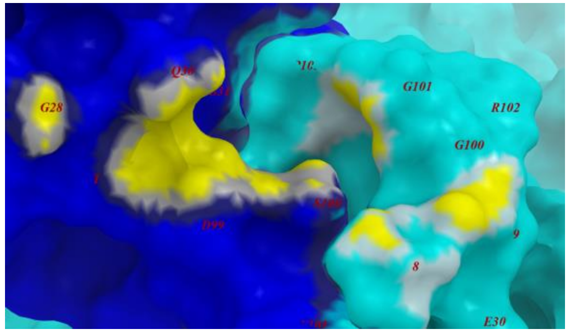

FIRMI 2

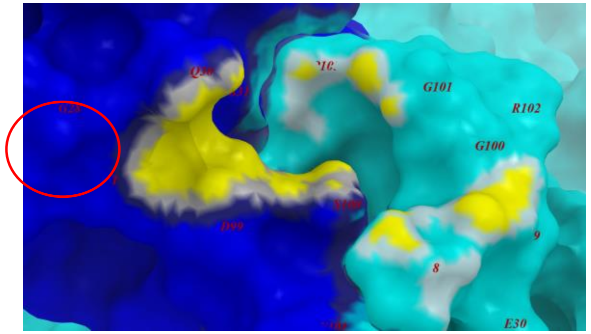

FIRMI 3

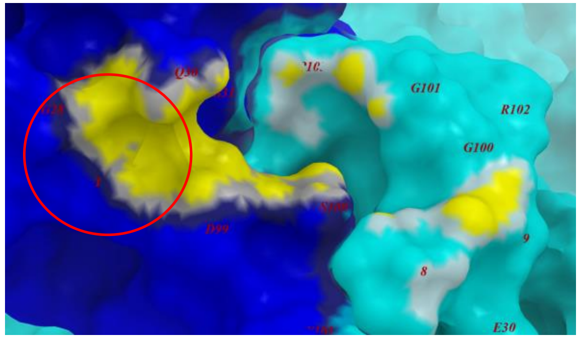

FIRMI 4

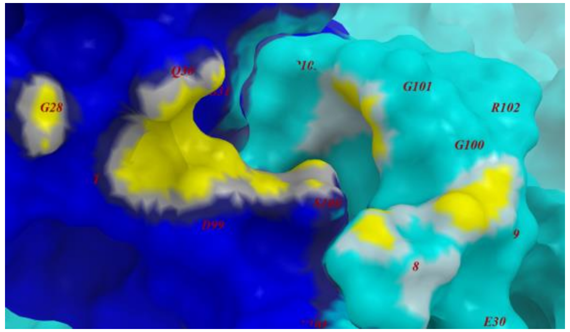

FIRMI 5

Suppl. Fig S6

MAGE-A3/12

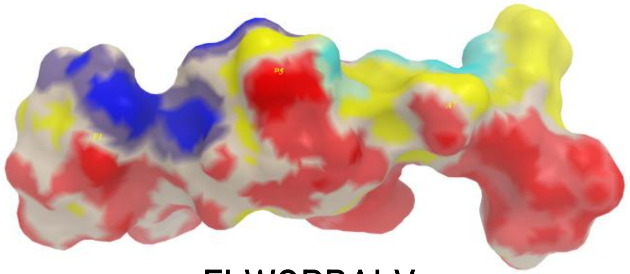

FLWGPRALV

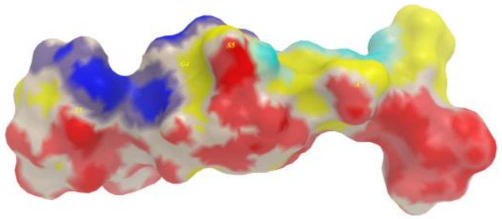

BACTO 1
FLWGSIALV

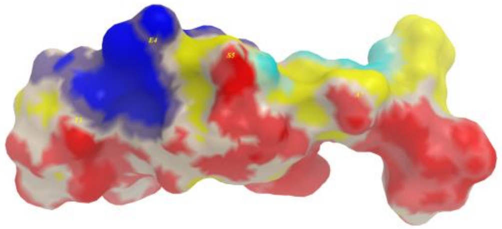

BACTO 2
FLWESRALV

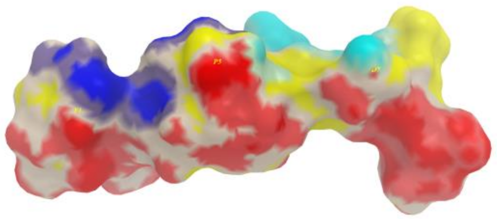

BACTO 3
FLWGPHDLV

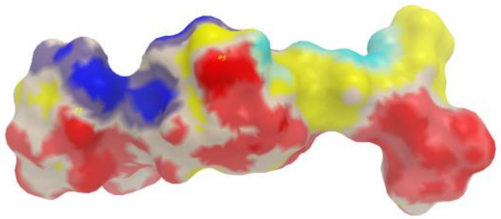

BACTO 4
FLWGPIGLV

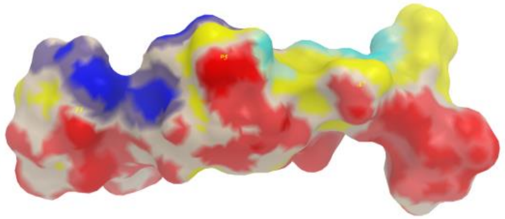

BACTO 5
FLWGPKALV

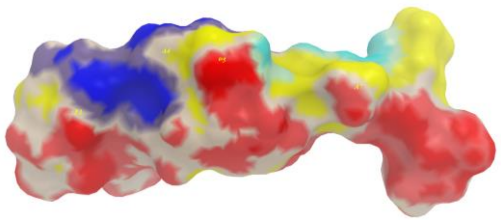

FIRMI 1
FLWAPIALV

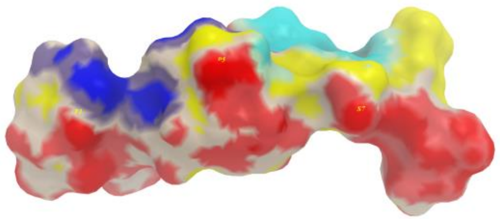

FIRMI 2
FLWGPFSLV

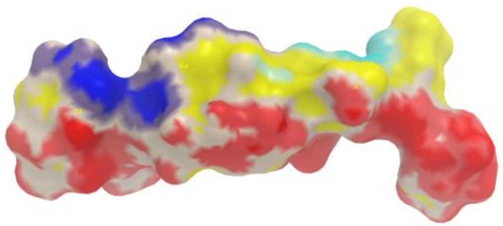

FIRMI 3
FLWGGRALL

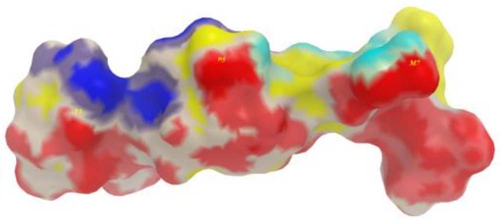

FIRMI 4
FLWGPVMLV

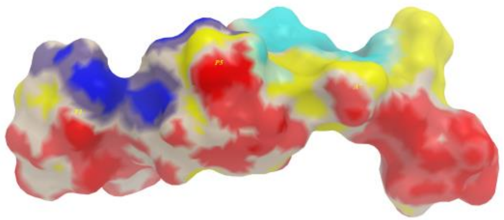

FIRMI 5
FLWGPFALI

Suppl. Fig S7

MAGE-A3/12

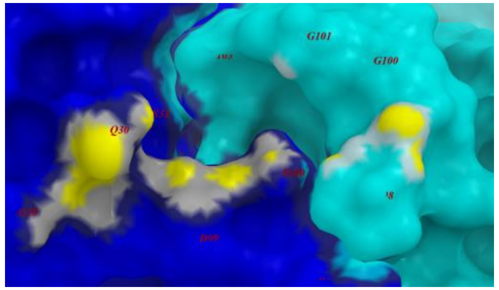

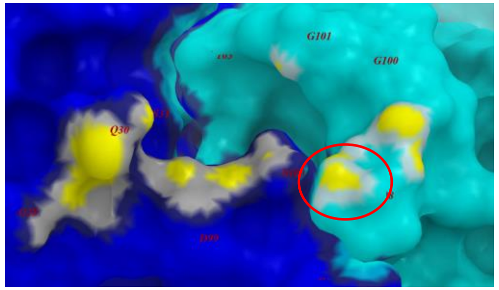

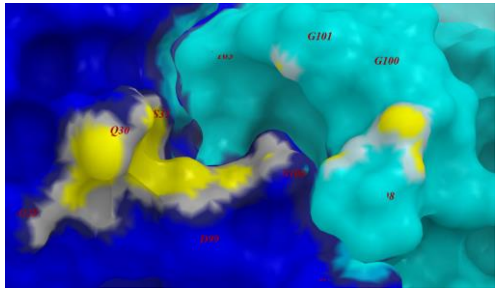

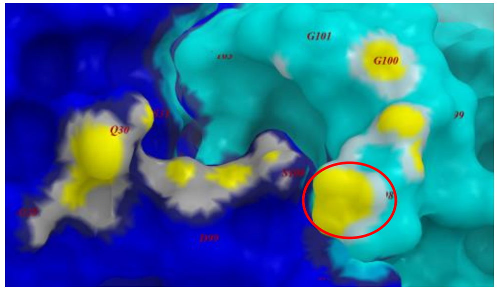

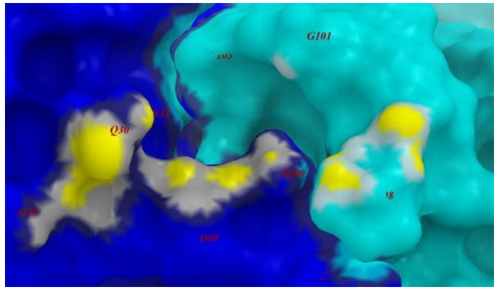

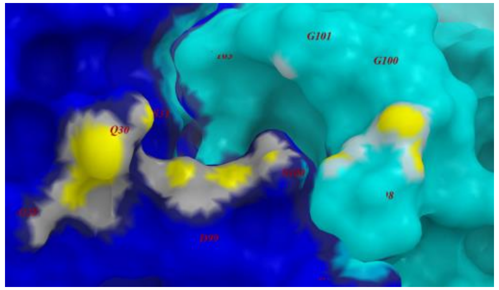

BACTO 1 | BACTO 2 | BACTO 3 | BACTO 4 | BACTO 5

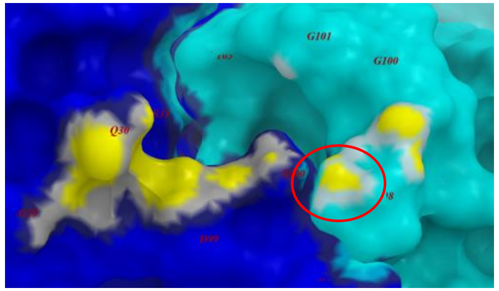

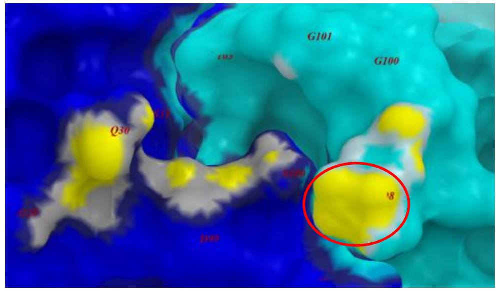

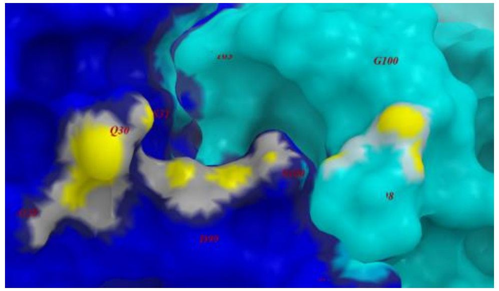

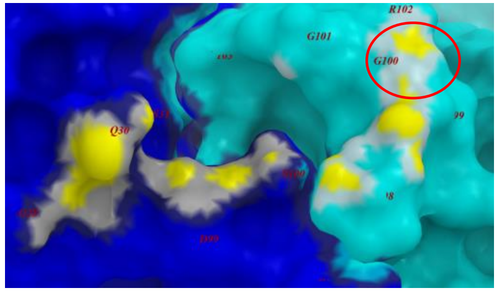

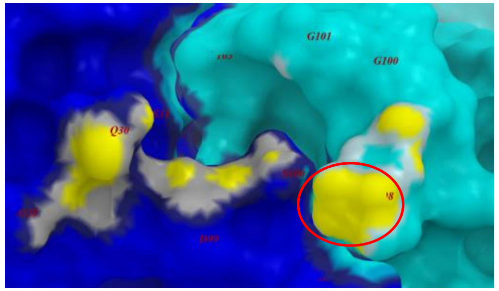

FIRMI 1 | FIRMI 2 | FIRMI 3 | FIRMI 4 | FIRMI 5

Suppl. Fig S8

MAGE-A10

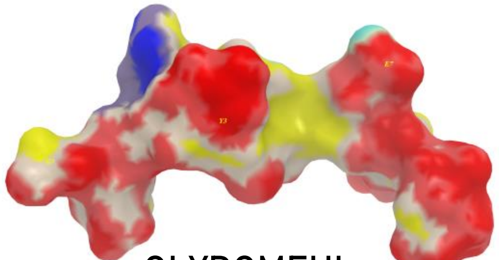

GLYDGMEHL

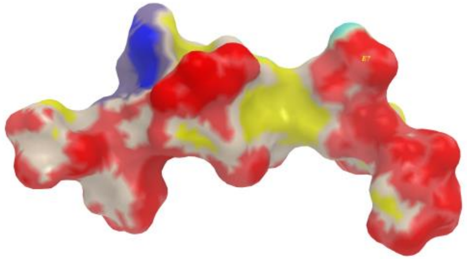

BACTO 1

ALYDGMEDL

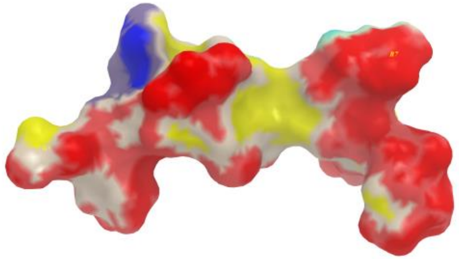

BACTO 2

GLYDGMRHL

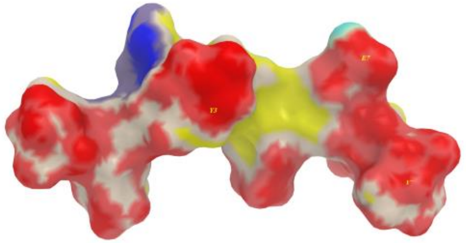

BACTO 3

VLYDGIEHV

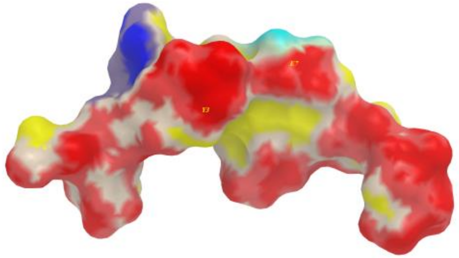

BACTO 4

GLYDGLENL

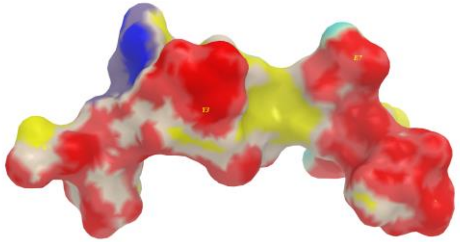

FIRMI 1

GLYDGMEYI

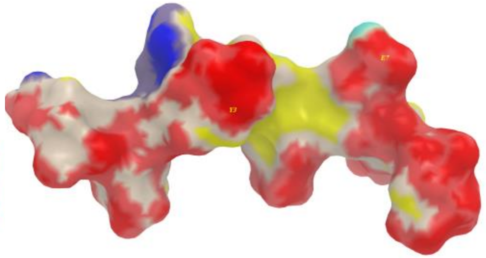

FIRMI 2

KLYDGIEHL

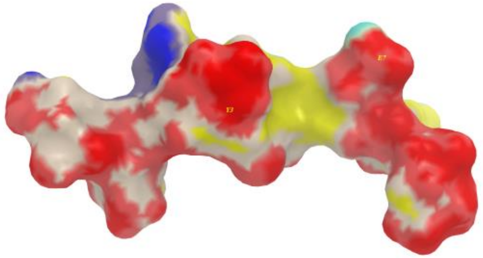

FIRMI 3

MLYDGMERL

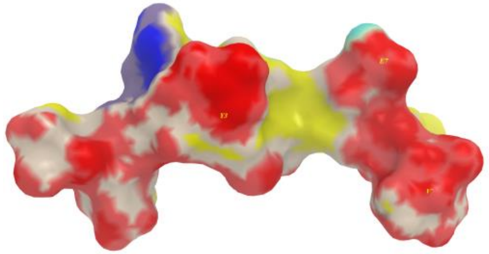

FIRMI 4

ALYDGMEEV

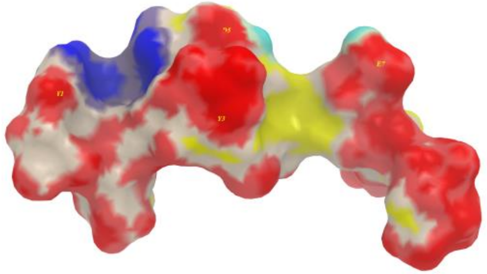

FIRMI 5

YLYDDMEHL

Suppl. Fig S9

MAGE-A10

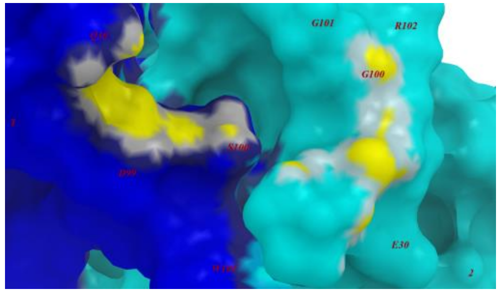

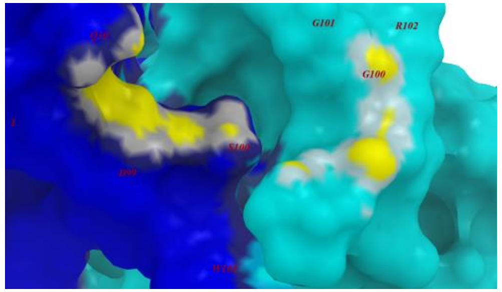
BACTO 1

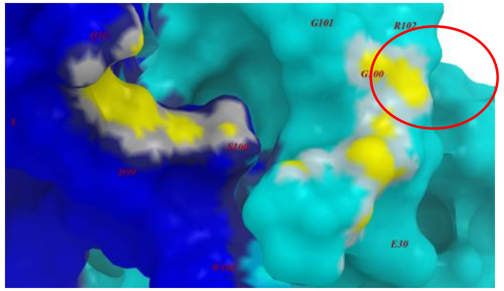
BACTO 2

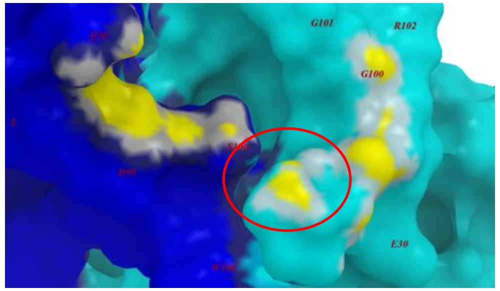
BACTO 3

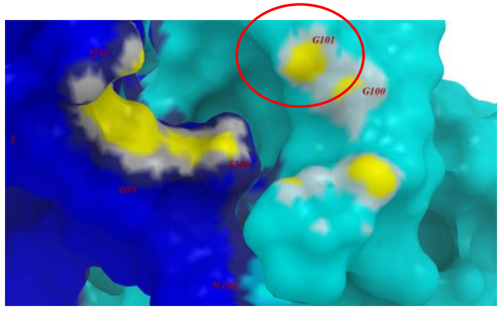
BACTO 4

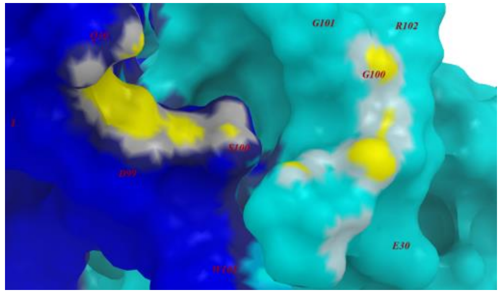
FIRMI 1

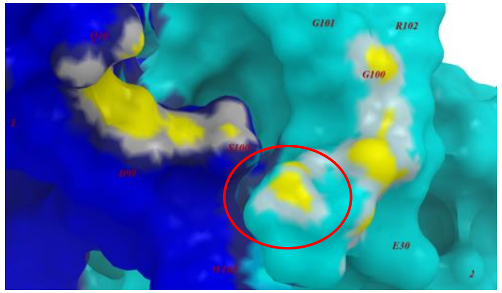
FIRMI 2

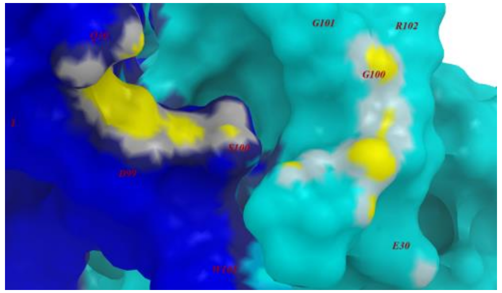
FIRMI 3

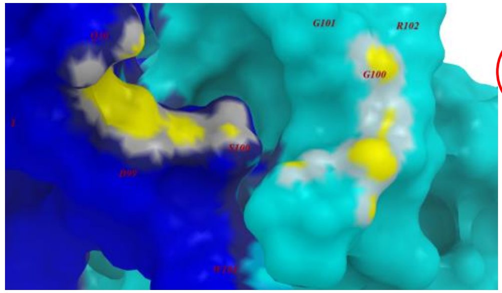
FIRMI 4

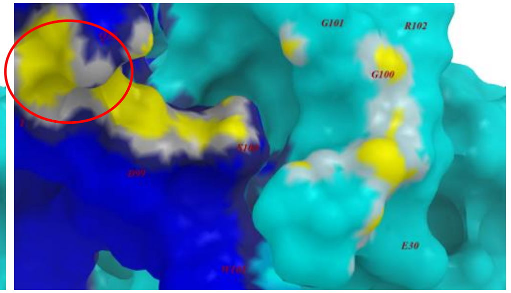
FIRMI 5

Suppl. Fig S10

MAGE-C1

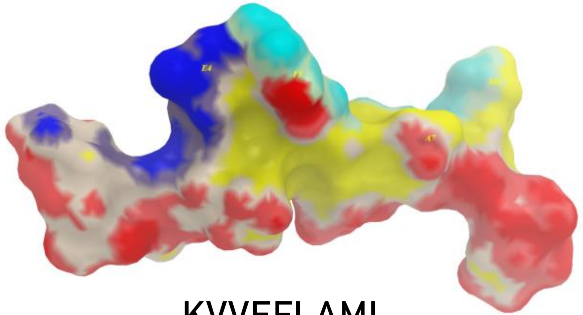

KVVEFLAML

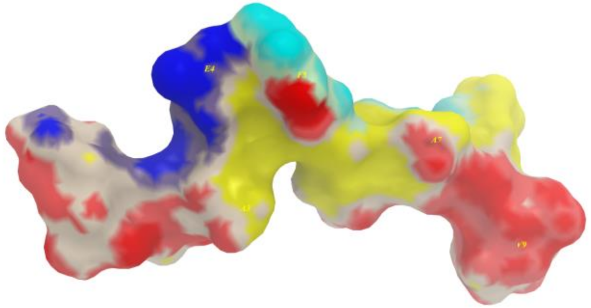

BACTO 1

KVAEFVALV

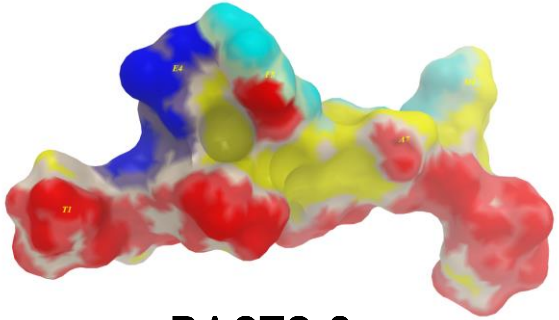

BACTO 2

TVLEFLAML

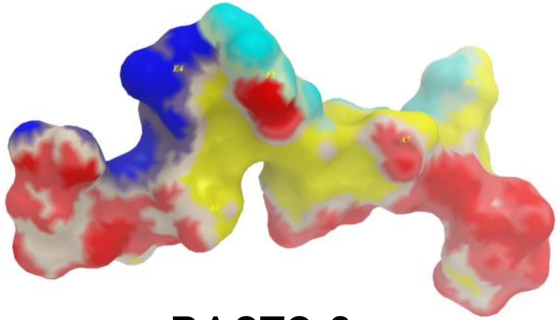

BACTO 3

QVAEFLAML

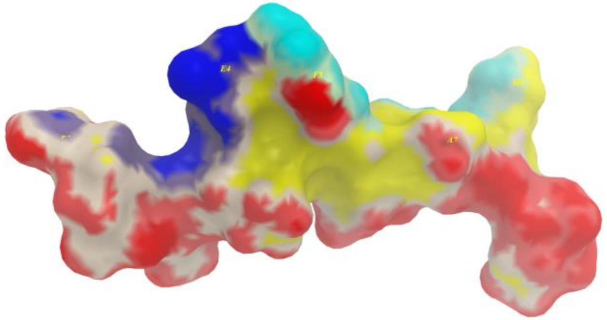

FIRMI 1

RLVEFLAML

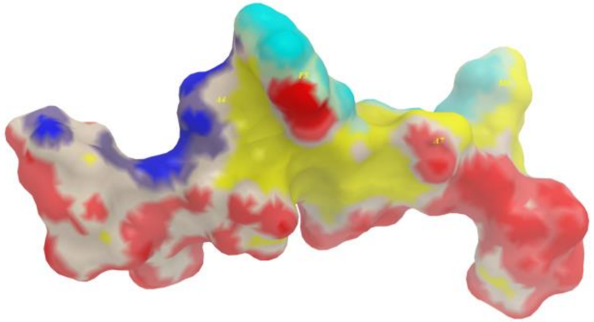

FIRMI 2

KLVAFLAML

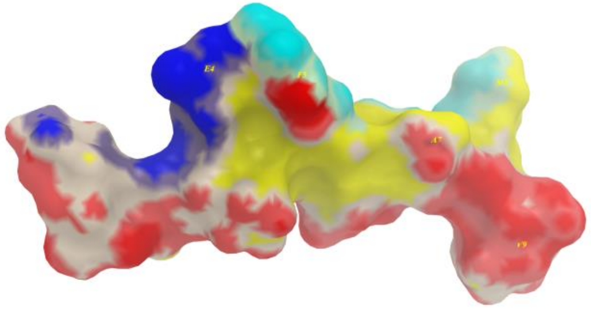

FIRMI 3

KTVEFLAMV

Suppl. Fig S11

MAGE-C1

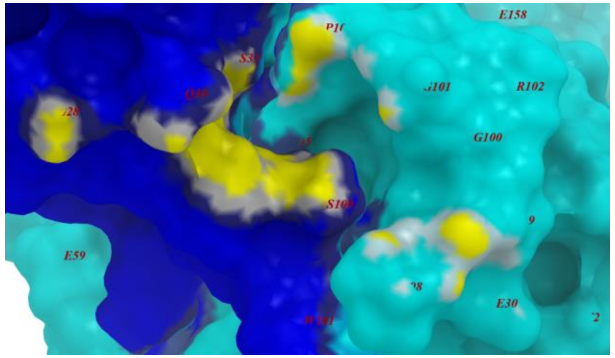

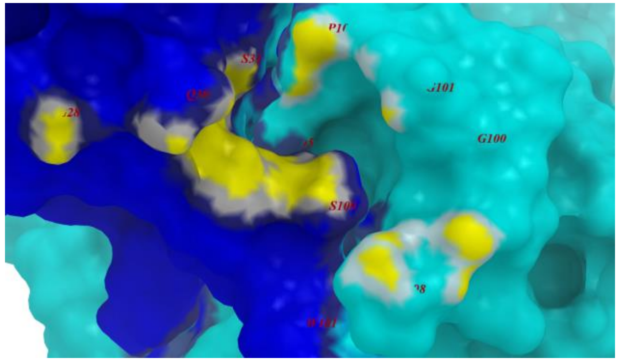

BACTO 1

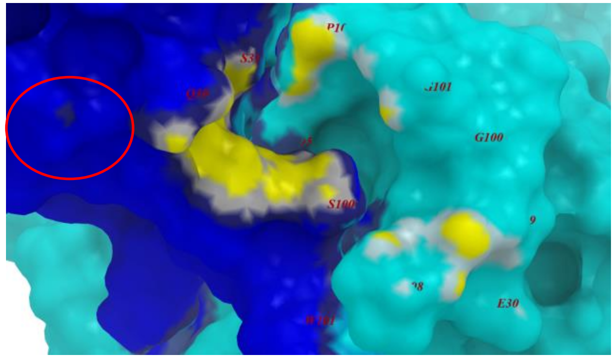

BACTO 2

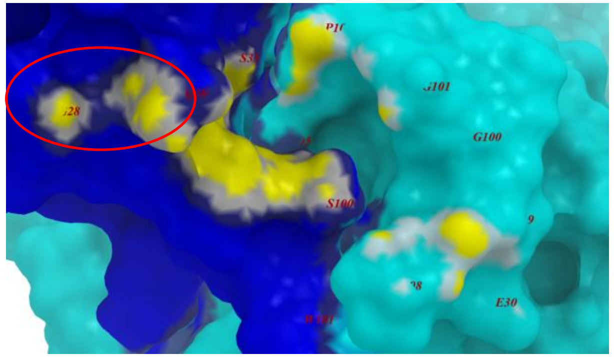

BACTO 3

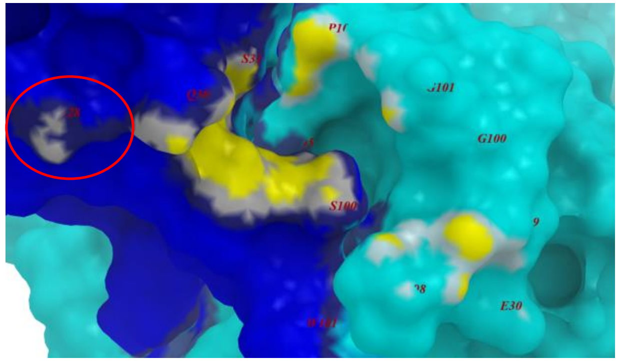

FIRMI 1

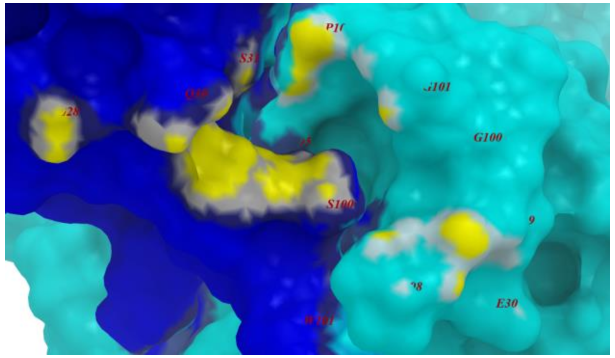

FIRMI 2

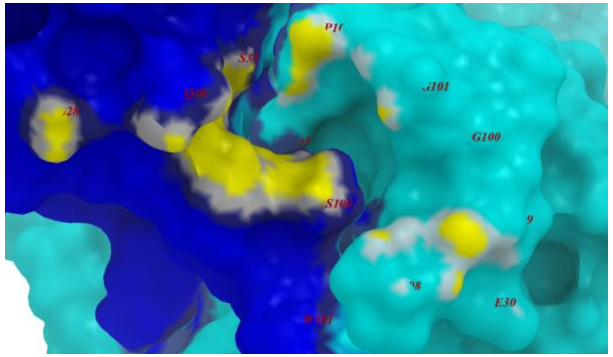

FIRMI 3

Suppl. Fig S12

MAGE-C2

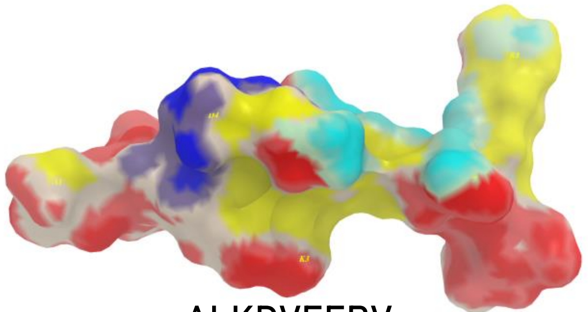

ALKDVEERV

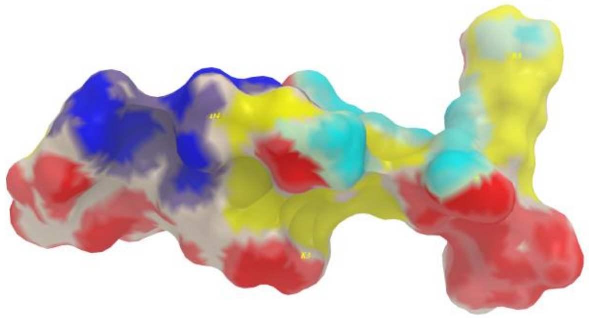

BACTO 1

YLKDVEERV

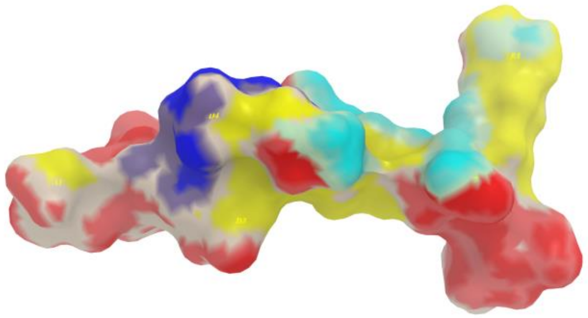

BACTO 2

ALDDVEERV

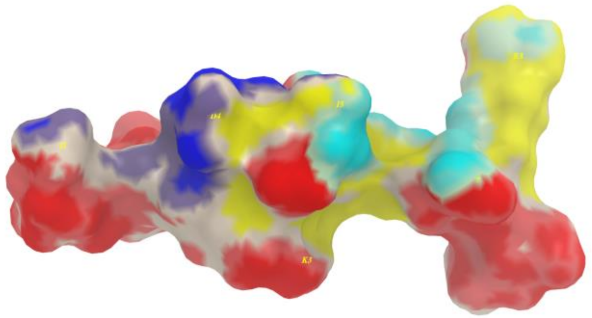

BACTO 3

ILKDIEERV

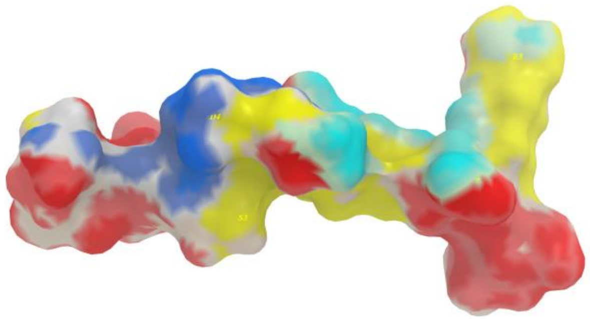

FIRMI 1

LLSDVEERV

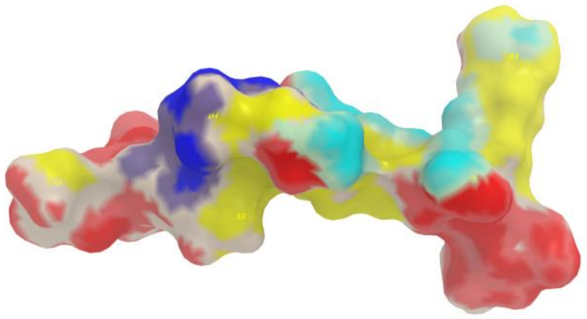

FIRMI 2

ALSDVEERV

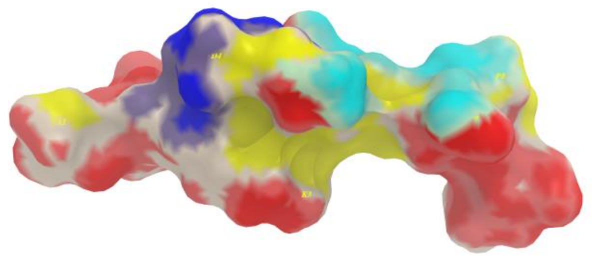

FIRMI 3

ALKDVEEPV

Suppl. Fig S13

MAGE-C2

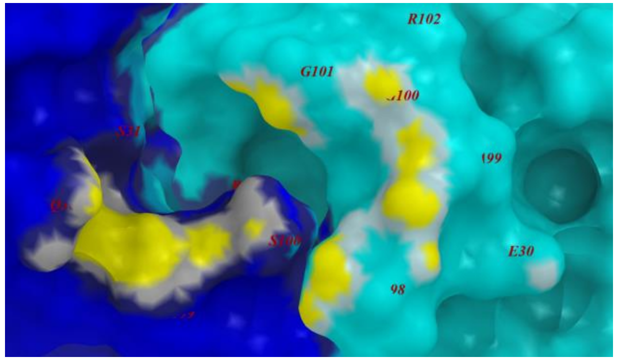

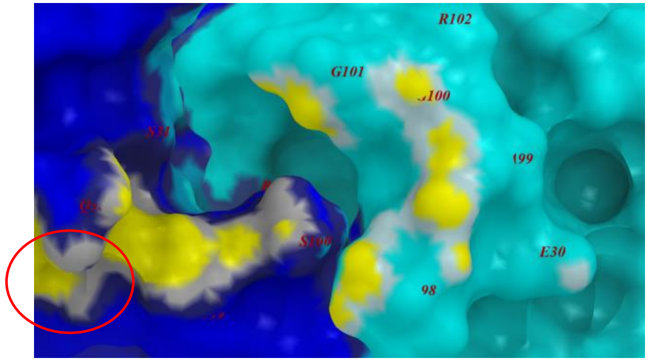

BACTO 1

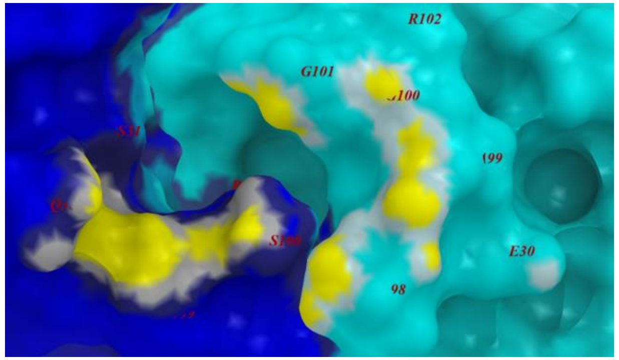

BACTO 2

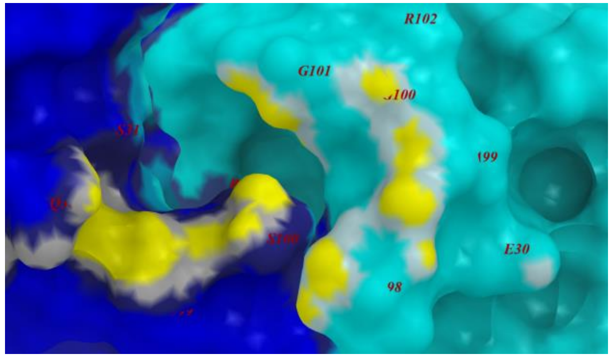

BACTO 3

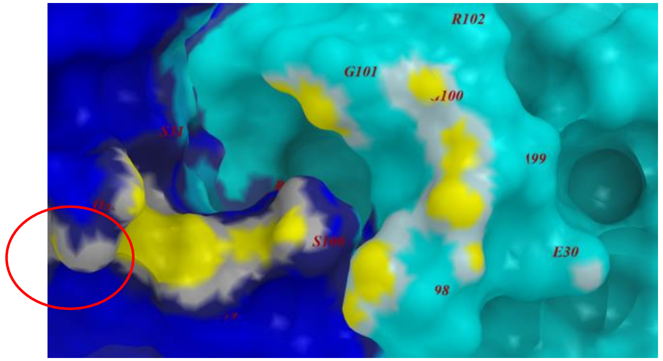

FIRMI 1

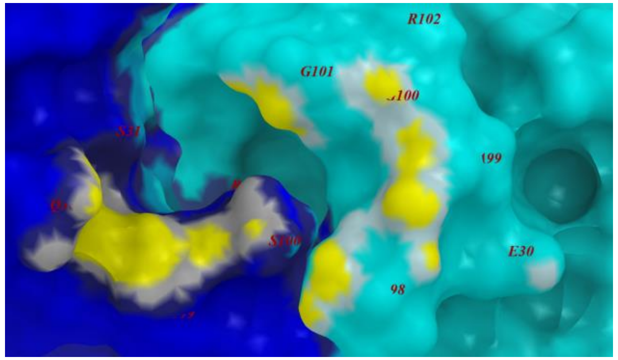

FIRMI 2

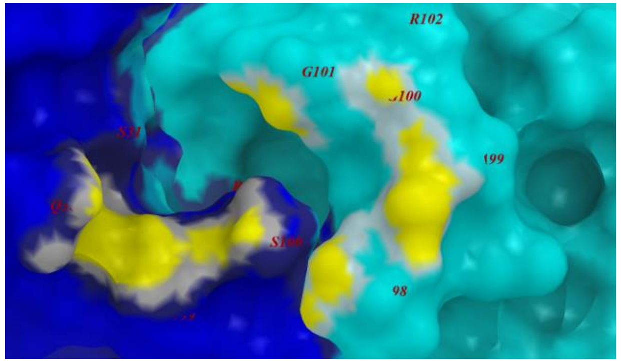

FIRMI 3

Suppl. Fig S14

SSX-2

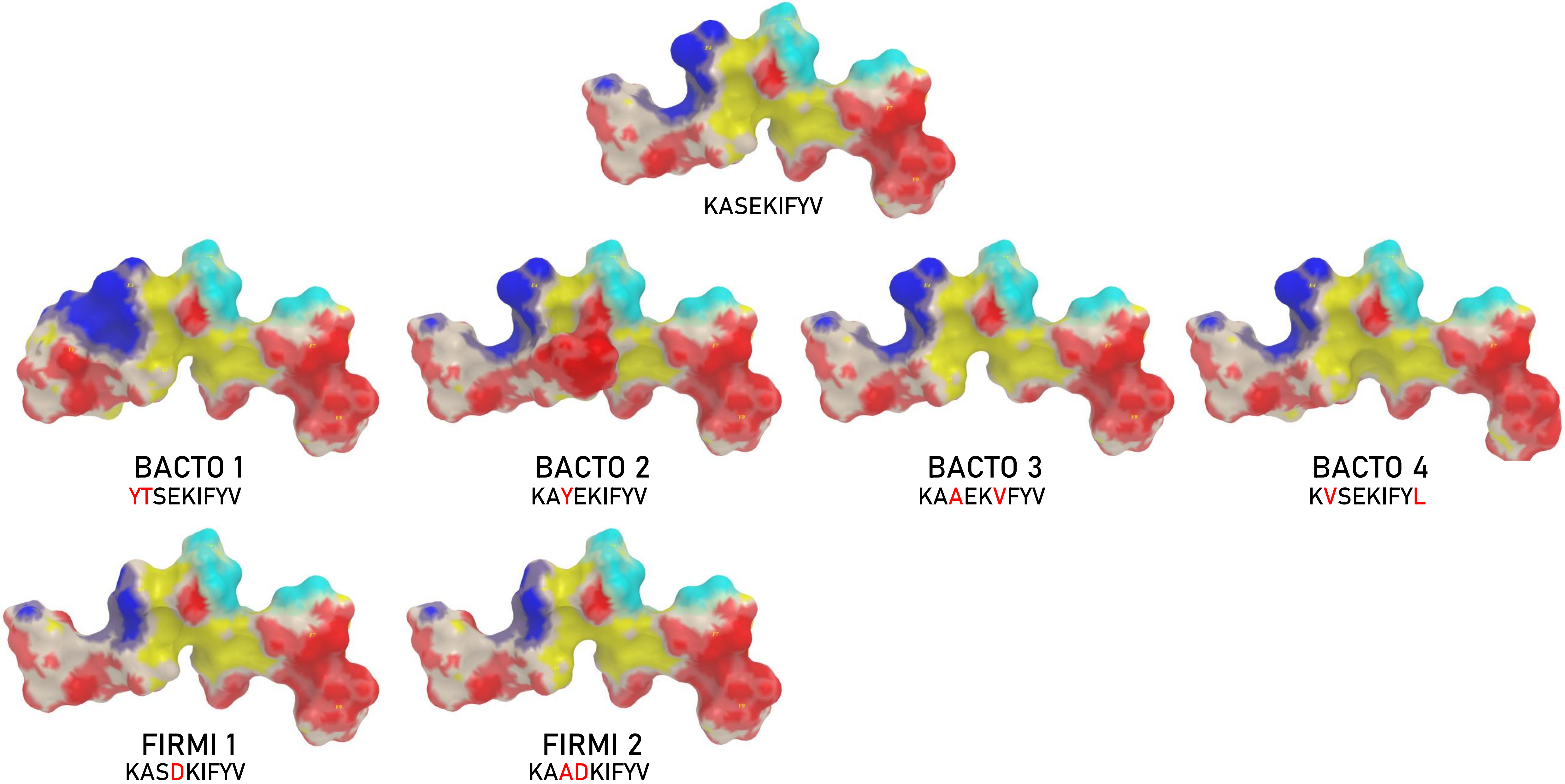

Suppl. Fig S15

SSX-2

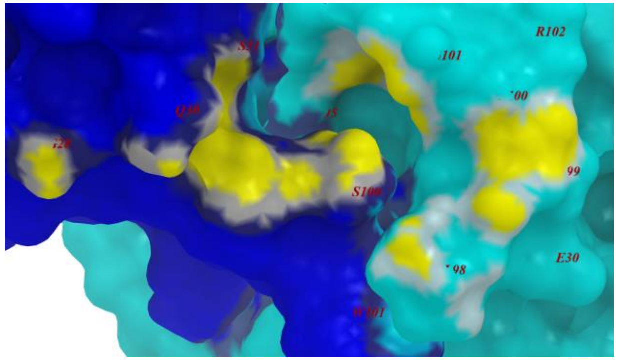

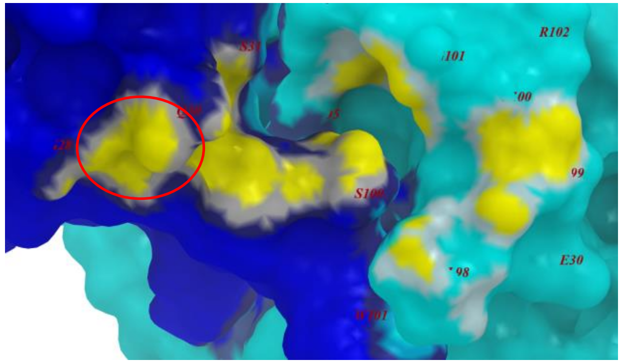

BACTO 1

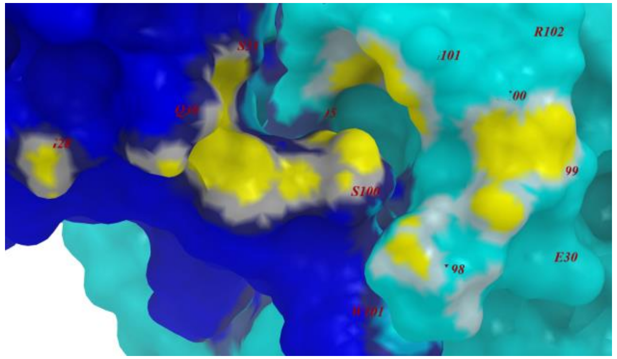

BACTO 2

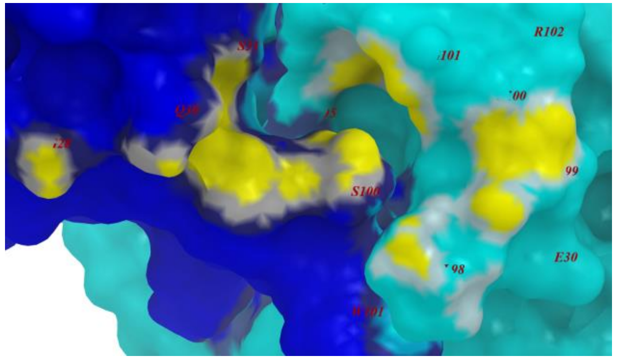

BACTO 3

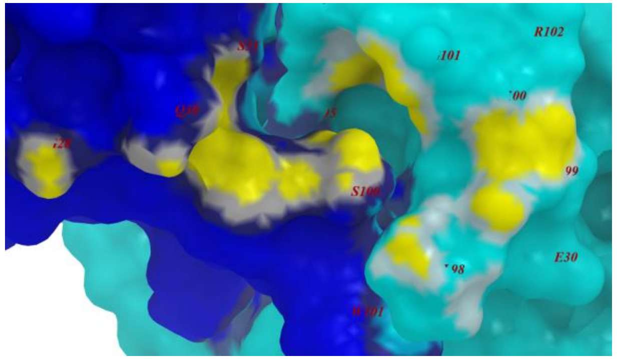

BACTO 4

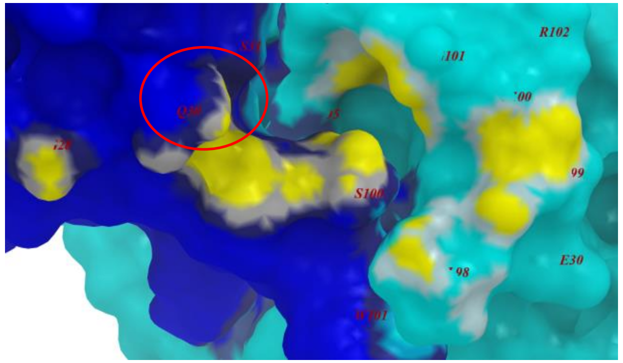

FIRMI 1

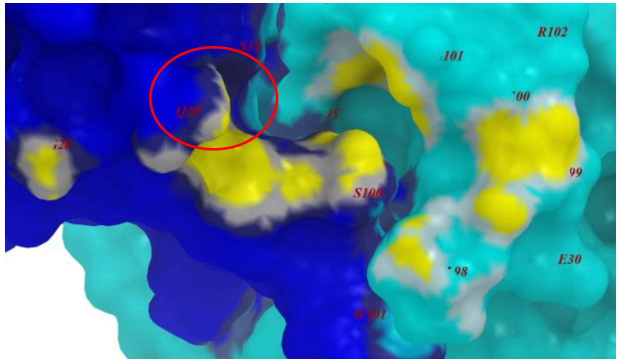

FIRMI 2

Suppl. Fig S16

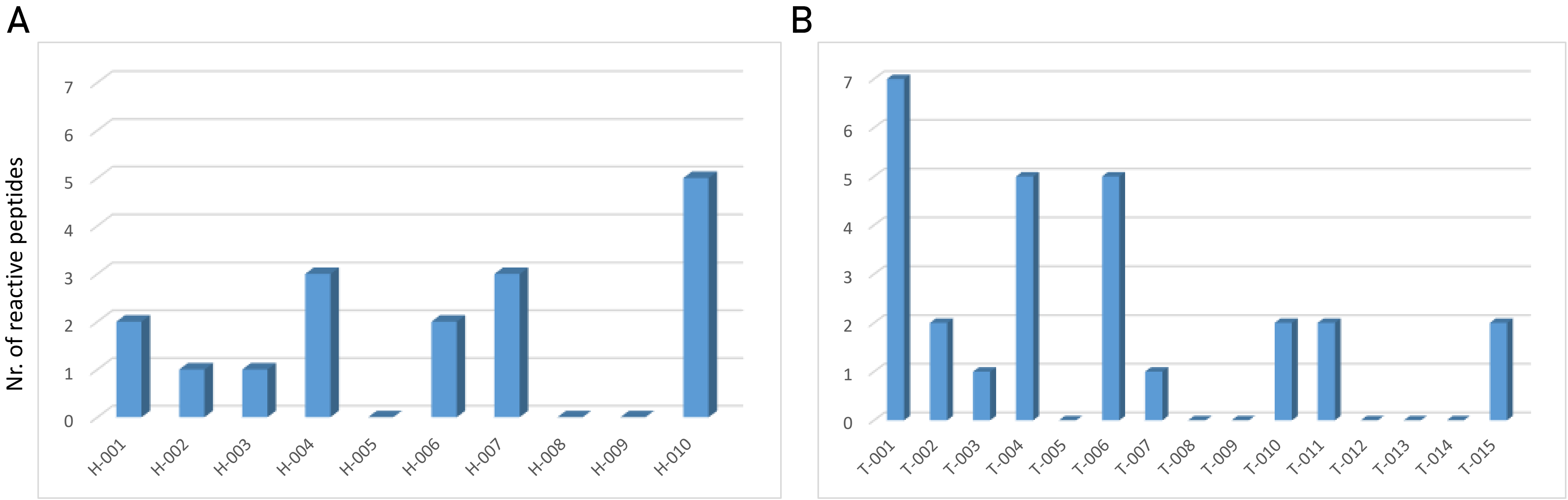

Supplement: Supplementary file 1 — Additional file 1: Suppl. Fig. S1. Descriptive flow cytometry plots for gating strategy on healthy donors and cancer patients’ PBMCs stained with DNA-barcoded pMHC multimers and surface antibody markers to sort viral/microbiome-derived (APC) and TAA/double positive (PE/PE+APC) multimer+ CD8+ T cells and to quantify multimer+ CD8+ T cells. Suppl. Figs. S2-15. Predicted 3D conformations of TAA and microbiota-derived paired peptides. The surface conformation of the paired TAA MoA-derived peptides is shown. Residues in the Mo epitopes that differed from the TAA sequences are indicated in red. A-Red areas = contact points with HLA-A molecule; blue areas = contact points with TCR α chain; Light Blue areas = contact points with TCR β chain. The images below the peptides show contact sites with the TCR (yellow areas). Suppl. Fig. S16. Diagrams show the total number of significant responses in DNA-barcoded pMHC multimers for HS (A) and CP (B). [file 13046_2024_3004_MOESM1_ESM.pdf]
